# Versatile self-assembled electrospun micropyramid arrays for high-performance on-skin devices with minimal sensory interference

**DOI:** 10.1038/s41467-022-33454-y

**Published:** 2022-10-03

**Authors:** Jia-Han Zhang, Zhengtong Li, Juan Xu, Jiean Li, Ke Yan, Wen Cheng, Ming Xin, Tangsong Zhu, Jinhua Du, Sixuan Chen, Xiaoming An, Zhou Zhou, Luyao Cheng, Shu Ying, Jing Zhang, Xingxun Gao, Qiuhong Zhang, Xudong Jia, Yi Shi, Lijia Pan

**Affiliations:** 1 Collaborative Innovation Center of Advanced Microstructures, School of Electronic Science and Engineering, Nanjing University, Nanjing, 210093 China; 2 Key Laboratory of Hydrology Water Resources and Hydraulic Engineering, Hohai University, Nanjing, 210098 China; 3 Shanxi Provincial People’s Hospital, Taiyuan, 030012 China; 4 School of Chemistry and Chemical Engineering, Nanjing University, Nanjing, 210093 China; 5 School of Materials and Metallurgy, Inner Mongolia University of Science and Technology, Baotou, 014010 China; 6 School of Chemistry and Chemical Engineering, Inner Mongolia University of Science and Technology, Baotou, 014010 China; 7 School of Physics, Nanjing University, Nanjing, 210093 China

**Keywords:** Materials for devices, Energy harvesting, Synthesis and processing

## Abstract

On-skin devices that show both high performance and imperceptibility are desired for physiological information detection, individual protection, and bioenergy conversion with minimal sensory interference. Herein, versatile electrospun micropyramid arrays (EMPAs) combined with ultrathin, ultralight, gas-permeable structures are developed through a self-assembly technology based on wet heterostructured electrified jets to endow various on-skin devices with both superior performance and imperceptibility. The designable self-assembly allows structural and material optimization of EMPAs for on-skin devices applied in daytime radiative cooling, pressure sensing, and bioenergy harvesting. A temperature drop of ~4 °C is obtained via an EMPA-based radiative cooling fabric under a solar intensity of 1 kW m^–2^. Moreover, detection of an ultraweak fingertip pulse for health diagnosis during monitoring of natural finger manipulation over a wide frequency range is realized by an EMPA piezocapacitive-triboelectric hybrid sensor, which has high sensitivity (19 kPa^−1^), ultralow detection limit (0.05 Pa), and ultrafast response (≤0.8 ms). Additionally, EMPA nanogenerators with high triboelectric and piezoelectric outputs achieve reliable biomechanical energy harvesting. The flexible self-assembly of EMPAs exhibits immense potential in superb individual healthcare and excellent human-machine interaction in an interference-free and comfortable manner.

## Abstract (web summary)

Designing efficient on-skin devices with minimal sensory interference remains challenging. Here, Zhang et al. develop versatile electrospun micropyramid array-based on-skin devices, achieving both imperceptibility and high performances in pressure sensing, radiative cooling, and bioenergy harvesting.

## Introduction

On-skin devices are functional patches or cloths attached to human skin to detect physiological and action signals, prevent injury to the body, convert bioenergy into electricity, etc^1–11^. These devices have shown great potential for applications in healthcare, behavior monitoring, individual protection, self-powered electronics, and human-machine interaction^12–22^. With the overwhelming evolution of these fields, on-skin devices are further required to achieve comfortable long-term use with diminished sensory interference. They are even expected to detect action and touch in natural states with minimal loss of touch sensation for more sophisticated applications, such as machine learning of a craftsman’s skills and restoration of limb function^5^. Therefore, great attention has been given to developing a variety of on-skin devices that not only avoid bringing discomfort to human skin but also cause minimal interference in the normal sense of touch.

Recently, Someya’s group proposed that ultrathin, ultralight, gas-permeable films are ideal candidates for imperceptible on-skin devices^5^. Limited by current technologies, especially the random spinning-deposition manner of electrospinning, the functional surfaces of reported imperceptible films are flat planes^5,23,24^. Because of the inferior optical, thermal, mechanical, and electrical properties of flat planes, endowing imperceptible on-skin devices with outstanding performance is difficult, restricting their application in many fields^9,10,25,26^. In contrast, three-dimensional (3D) microstructure arrays with gradient geometries, for example, micropyramid, microcone, microdome, and microprism arrays, have an edge in developing high-performance devices. The reason is that their inherent features of gradient space filling, gradient stress distribution, and gradient refractive index are beneficial for regulating force, heat, light, and electricity^8–10,13,25–28^. However, existing 3D microarray processing technologies such as photolithography and 3D printing fail to simultaneously realize high gas permeability, ultralow thickness, ultralight weight, and gradient geometry^8,10,13,25–27,29–34^. The challenge, consequently, is to create a new type of 3D microstructure array with both a gradient geometry and a gas-permeable, ultrathin, ultralight structure to provide various imperceptible on-skin devices with superior performance.

**Fig. 1:**
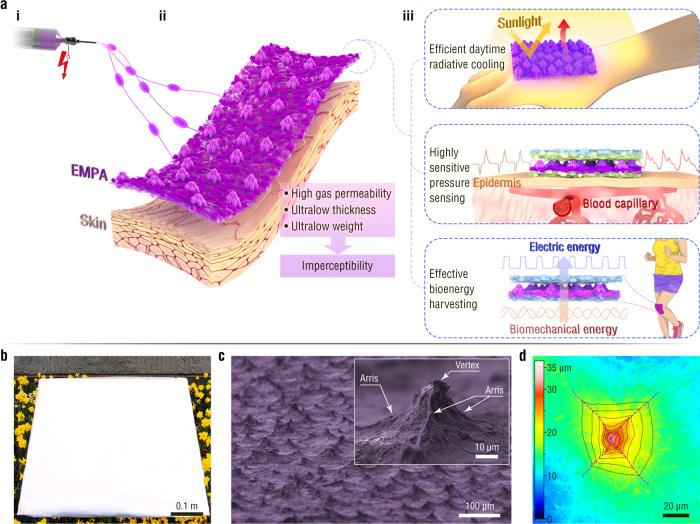
Material structure design. **a** Schematic illustration of the (i) fabrication, (ii) structure and (iii) application of EMPAs. **b** Photograph of a large-area EMPA-based film. **c** SEM image of an EMPA. The inset shows a magnified SEM image of an electrospun micropyramid. **d** Laser confocal microscopy (LCM) image of an electrospun micropyramid. The black dotted lines and the purple dashed lines are isohypses and arrises of the electrospun micropyramid architecture, respectively.

Here, we construct unique, ultrathin, ultralight, gas-permeable electrospun micropyramid arrays (EMPAs) by electrospinning self-assembly to endow imperceptible on-skin devices with excellent performance in various applications (Fig. 1). A series of wet heterostructured electrified jets can be assembled into structurally designable EMPAs made from various materials. Benefiting from flexible designability, the optimal optical, thermal, mechanical, and electrical properties of EMPAs are exploited to achieve outstanding performance for imperceptible on-skin devices applied in daytime radiative cooling, pressure sensing, and biomechanical energy harvesting. The high visible to near-infrared (vis-NIR) reflectivity (97.9%) and mid-infrared (MIR) emissivity (76.3%) of an EMPA-based radiative cooling fabric as thin as 47 µm reduce the temperature by ~4 °C under a solar intensity of 1 kW m^–2^. Moreover, an EMPA piezocapacitive-triboelectric hybrid sensor with the advantages of high sensitivity (19 kPa^−1^), ultralow detection limit (0.05 Pa), and ultrafast response can detect an ultraweak fingertip pulse for health diagnosis during monitoring of natural finger manipulation over a wide frequency range. In addition, the high triboelectric and piezoelectric outputs (105.1 µC m^−2^) of EMPA nanogenerators enable effective biomechanical energy harvesting. Our findings open an upgradable way for developing next-generation on-skin devices with both superior performance and imperceptibility to meet the high-level requirements in multiple application scenarios.

## Results

### Architecture, growth process, and designability of EMPAs

**Fig. 2:**
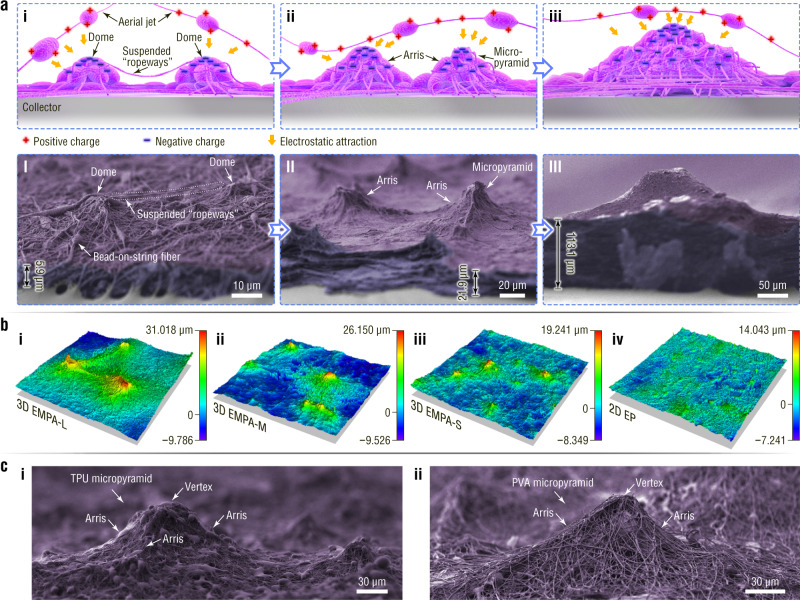
Growth process and structural and material designability of EMPAs. **a** Schematic illustration and SEM images showing the growth process of EMPAs. **b** LCM images of EMPA-based films with average pyramid heights of (i) 24.75, (ii) 18.23, and (iii) 11.37 μm and (iv) a flat electrospun film. **c** SEM images of (i) TPU and (ii) PVA micropyramids.

Electrospun pyramid arrays with unique architecture are self-assembled using a far-field electrospinning apparatus (Fig. 1a-i) with electrically grounded aluminium foil as a collector (Supplementary Fig. 1). Poly(vinylidene fluoride) (PVDF) is first chosen as a material model for the proof-of-concept study. A large-area EMPA-based PVDF film (0.45 × 0.58 m^2^) is successfully fabricated via this single-step self-assembly (Fig. 1b). The micropyramids are well distributed on the sample surface (Fig. 1c), and the structural unit is in accordance with the typical features of a pyramid in which sloping triangular sides meet at a vertex (inset in Fig. 1c and d)^35^. The arrises of the pyramids, in general, appear along the connecting lines of two adjacent vertices. The number of arrises ranges from three to six for a single electrospun micropyramid (Supplementary Fig. 3a). In contrast to micropyramids prepared via previous technologies such as photolithography and 3D printing^8,10,13,25–27,29–31^, the electrospun micropyramids have a gas-permeable network structure consisting of bead-on-string micro/nanofibers (Fig. 2a-I and Supplementary Fig. 3b, c).

Figure 2a shows the growth process of EMPAs, which is deduced from scanning electron microscopy (SEM) images showing the dependence between the thickness and morphology. The initial deposition of wet heterostructured electrified jets leads to the formation of inhomogeneously charged microdomains^36,37^. Due to the electrostatic interaction^38,39^, the positively charged aerial jets tend to be deposited on the negatively charged microdomains with higher PVDF contents. Subsequently, these microdomains develop into fibrous domes (i.e., embryonic forms of EMPAs) when the substrate thickness (i.e., the thickness of the fibre mat other than the electrospun pyramid array) reaches several microns (Fig. 2a-I)^40,41^. A detailed discussion is presented in Supplementary Fig. 4, Note 1–3. Meanwhile, electrostatic induction and polarization from the electrostatic field endow the dome tops with negative charges (Fig. 2a-i), and the position closer to the tip of a protuberance possesses a higher local electric field^36,40–43^ (see evidence in Supplementary Note 4). Therefore, there are always single fibers crossing the tops of neighboring domes, forming suspended “ropeways” (Fig. 2a-i and dotted area in Fig. 2a-I). The suspended “ropeways” act as frameworks to catch subsequent aerial jets, outlining the arrises of the architecture. Meanwhile, microdome arrays evolve into prominent EMPAs when the substrate thickness exceeds 20 μm (Fig. 2a-ii, a-II).

As the electrospinning self-assembly continues, the initially formed EMPA further grows in size. Adjacent micropyramids with similar heights enlarge and even further fuse into larger pyramids with the increase in deposited fibers (Fig. 2a-iii, a-III, Supplementary Fig. 3d, e). The SEM image in the dashed box of Supplementary Fig. 3e-iv shows an intermediate state of fusion, i.e., multiple vertexes appearing on a single protuberance. For adjacent micropyramids with different heights, short micropyramids with low growth speed are submerged in the gradually thickening substrate. Therefore, the interval of the micropyramid vertexes increases with increasing substrate thickness (Supplementary Fig. 3f). The self-assembled electrospun pyramids can grow from the micron to millimeter scale.

The self-assembly technology is flexible for the structural and material design of EMPAs. In terms of structural designability, first, the electrospinning voltage can dominate the growth of EMPAs. As the voltage increases from 10.0 to 17.5 kV, the micropyramids first appear, then dwindle in size, and finally disappear (Supplementary Fig. 10a). At a voltage of 12.5 kV, the largest size of the structural unit for EMPAs is observed because the fibre size and the quantity ratio of beads to strings reach the best values (Supplementary Note 5). The second effective factor for structural control is humidity. Atmospheric water can help discharge the aerial jets and therefore decrease the charge density^44^. Generally, increased relative humidity in electrospinning reduces the size of electrospun micropyramids (Supplementary Fig. 10b). The third approach for structural control is adjusting the horizontal swing distance of the syringe to control the substrate thickness to obtain EMPAs with the desired sizes (Supplementary Fig. 10c). Relying on the above regulation approaches, films comprising large-, medium-, and small-sized 3D EMPAs (i.e., three types of EMPAs with different average micropyramid heights) are successfully fabricated (Supplementary Fig. 11). Correspondingly, they are designated 3D EMPA-L (Fig. 2b-i), 3D EMPA-M (Fig. 2b-ii), and 3D EMPA-S (Fig. 2b-iii). The film with the two-dimensional (2D) electrospun plane structure is designated 2D EP (Fig. 2b-iv). Moreover, the self-assembly technology is adaptable in that various available materials can be processed into EMPAs, such as PVDF, thermoplastic polyurethane (TPU), and poly(vinyl alcohol) (PVA) (Fig. 2c).

### Imperceptibility of EMPAs and resultant on-skin devices

**Fig. 3:**
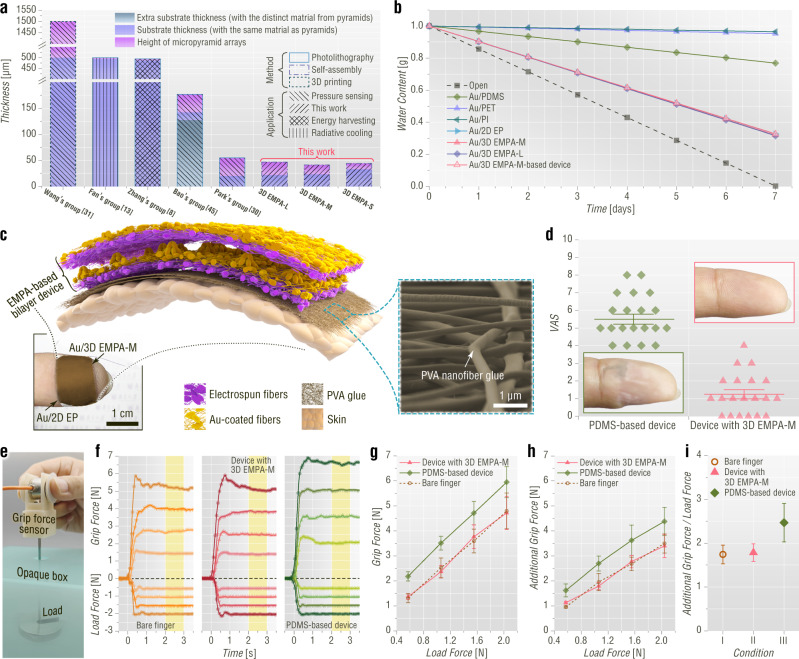
Imperceptibility evaluations of EMPA-based on-skin devices. **a** Plot for a comparative study of micropyramid-array-based flexible devices in terms of thickness. **b** Water vapor transmission tests of three different types of conventional on-skin film materials and EMPA-based on-skin devices. **c** Schematic diagram of the EMPA-based bilayer on-skin device. The inset shows an SEM image of the on-skin electrospun PVA nanofiber glue. **d** Participants reported any feelings while two types of devices were attached to the fingertip, which was evaluated based on a VAS (0–10). Crosses indicate the mean, and error bars are the standard error of the mean. The insets in the green and pink boxes show fingertip skin after being covered with different on-skin devices for seven hours. **e** Photograph showing the measurement of the grip force with different devices attached to the index finger. **f** Simultaneous grip force and load force of a participant with and without different devices attached to the finger. The yellow-shaded area shows the time over which the grip force was measured for analysis. **g** Grip force scaled with the load force across all participants. Error bars are the standard deviations. **h** Friction-adjusted additional grip force scaled with the load force across all participants. Error bars are the standard deviations. **i** Ratio of additional grip force to load force across all participants for different conditions. Error bars are the standard deviations.

The 3D EMPA-L, 3D EMPA-M, 2D EP, and EMPA device comprising a 3D EMPA-M and a 2D EP are chosen for the comparative study of imperceptibility. Obviously, the self-assembled EMPA films with thicknesses no more than 50 μm are ultrathin compared with previously reported micropyramid array films (Fig. 3a)^8,10,13,30,31,45^. Moreover, the EMPA films are ultralight, with a mass per square centimeter of only 1.1 mg. Furthermore, the EMPA films composed of loose micro/nanofibers display excellent gas permeability to facilitate skin breathing, as evaluated by the water weight loss method^3,4^ (Supplementary Fig. 14a–c). Compared with traditional on-skin materials—such as gold (Au)-coated 300 µm-thick polydimethylsiloxane (PDMS), 120 µm-thick polyethylene terephthalate (PET), and 50 µm-thick polyimide (PI) films—the water weights of the bottles covered by Au-coated EMPA films decrease at ultrahigh rates (~0.55 kg m^−2^ d^−1^, Fig. 3b).

The imperceptibility of the on-skin EMPA bilayer device is evaluated based on a visual analog score (VAS) and an object-grasping experiment, with a conventional bilayer device composed of a 500 µm-thick pyramidal microstructure PDMS film and a polypropylene film as a reference (Supplementary Fig. 14e). The ultralight and ultrathin EMPA bilayer device with electrospun PVA nanofiber glue is easily laminated on the fingertip by humidification treatment (Fig. 3c and Supplementary Fig. 14f). In contrast, the heavy and thick PDMS-film-based bilayer device must be bound to a fingertip by tape. Seven hours later, the skin wearing the PDMS-film-based device becomes wrinkled and whitened. On the contrary, the skin under the EMPA bilayer device is fine and unchanged in appearance (Fig. 3d). Twenty participants report the feelings of sensation when wearing the devices on a VAS scale of 0–10 (0: no sensory interference, 10: extreme discomfort, Supplementary Table 3). 95% of the participants report that the EMPA device exerts no impact on work and daily life, whereas the conventional PDMS-film-based device does affect the work and daily life of all participants. The score of the EMPA device is 1.25, which is only 23% of the value for the PDMS-film-based device.

For the object-grasping experiment, eighteen participants grasp, lift and hold an instrumented object with adjustable masses by their thumb and index fingers (Fig. 3e) under different conditions: (i) bare finger, (ii) EMPA device, and (iii) conventional PDMS-film-based device. If the on-skin device affects the sensory feedback from the finger, then participants will produce a larger grip force for the same load force^46,47^. The grip force is quantified over the interval between 2 and 3 s after object lift-off (Fig. 3f), and its average value at each condition and load force is calculated (Fig. 3g). Furthermore, we also calculate the additional grip force in which the interaction between the finger and the object is removed (Fig. 3h and Supplementary Note 6). As shown in Fig. 3g–i, both grip forces and additional grip forces are similar between bare finger and EMPA device conditions but were larger for the PDMS-film-based device condition. Post hoc tests are used to further check. Conventional PDMS-film-based device exhibits significant differences compared with bare finger and EMPA device conditions (all comparisons *P* < 0.001). In contrast, the p-values of both grip force and additional grip force for bare finger and EMPA device conditions are 1.0, indicating that the on-skin EMPA device does not interfere with the sensorimotor processing of object grasping. Therefore, EMPA devices have excellent imperceptibility.

### Various applications of EMPA on-skin devices with high performance

The performance enhancement effects of micropyramids due to their gradient space-filling, gradient stress distribution, and gradient refractive index have been demonstrated, which bring about advanced optical, thermal, mechanical, and electrical properties^7–10,13,25–31,45,48–50^. Similarly, EMPA-based on-skin devices exhibit high performance in various application fields, such as daytime radiative cooling, pressure sensing, and bioenergy harvesting. 2D EP, 3D EMPA-S, 3D EMPA-M, and 3D EMPA-L were used to investigate the advancedness of the EMPA devices and their performance optimize ability due to the structural designability (Supplementary Note 7).

**Fig. 4:**
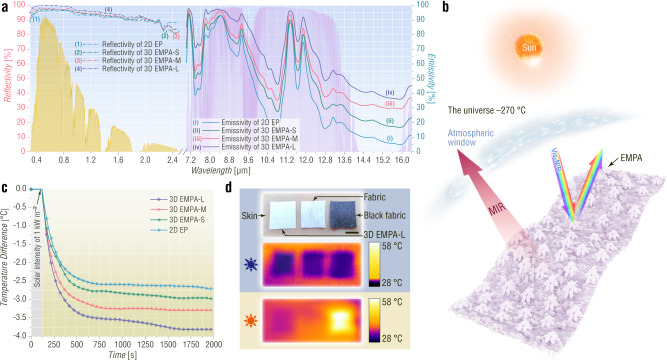
Optical properties and application of EMPAs in efficient daytime radiative cooling. **a** Spectral vis-NIR/MIR reflectance/emittance of the electrospun films presented against the AM1.5 solar spectrum (yellow shaded area) and the atmospheric transparency window (purple shaded area). **b** Schematic illustration of the working mechanism of the EMPA-based passive radiative cooling fabric. **c** Temporal temperature difference profile measured for electrospun-film-based radiative cooling fabrics. **d** Photograph and thermal camera images of the on-skin EMPA-based film, white cotton-containing fabric, and black cotton-containing fabric before and after solar irradiation for approximately 8 min. Scale bar, 5 mm.

Imperceptible EMPA films show an excellent daytime radiative cooling effect, which allows human body cooling without energy consumption^12,51–57^. For 2D EP, 3D EMPA-S, 3D EMPA-M, and 3D EMPA-L, as the average pyramid height (APH) increases from 0 to 24.75 μm, the MIR emissivity increases from 51.1% to 76.3%, and their vis-NIR reflectivities are close to each other (between 94.7% to 97.9%) (Fig. 4a and Supplementary Fig. 16a, b). Compared with 2D EP, the improved emissivity results from the gradient refractive index of EMPAs^11,13^. The spectrum data indicate that the PVDF EMPAs not only have low absorptance of sunlight in the vis-NIR region by Mie scattering^52^ but also can increase the thermal energy emitting to cold outer space through the transparent atmospheric spectral window (TASW) in the MIR region (Fig. 4b)^14^. Therefore, the 47 μm-thick 3D EMPA-L with the highest APH displays the best sub-ambient temperature drop of 3.86 °C under a solar intensity of 1 kW m^–2^ (Fig. 4c and Supplementary Fig. 16c–e). In practice, when the 3D EMPA-L is attached to human skin and exposed to sunlight with an intensity of 1 kW m^–2^ for 8 min, the skin can be cooled down 4 °C lower than that exposed to sunlight (Fig. 4d). In contrast, a white cotton-containing fabric possesses a poor cooling outcome (1.6 °C lower than the temperature of the skin exposed to sunlight) under the same test conditions. Moreover, a black cotton-containing fabric even severely increases the skin temperature (Supplementary Fig. 16f).

High-performance electronic skins and imperceptible operation are achieved with EMPA-based piezocapacitive sensors, triboelectric nanogenerators (TENGs), and piezoelectric nanogenerators (PENGs), opening the possibilities of sensitive force monitoring from weak pulses to powerful motions in natural states and effective bioenergy harvesting without discomfort. Both the piezocapacitive sensor (Supplementary Fig. 17a) and the TENG (Supplementary Fig. 18a) consist of an Au-coated electrospun film with different APHs and a Au-coated flat electrospun film. An in situ polarized electrospun PVDF film (Supplementary Fig. 21a, b) is sandwiched between two Au electrodes to form the PENG (Supplementary Fig. 21c).

**Fig. 5:**
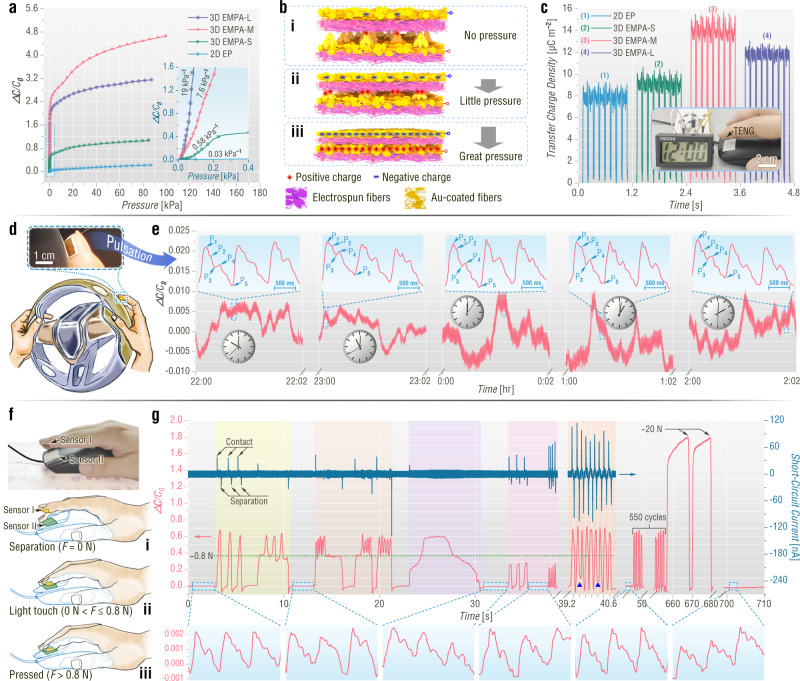
Electrical properties and applications of EMPAs in highly sensitive pressure sensing and effective bioenergy harvesting. **a** Relative capacitance change (*△C*/*C*_0_) as a function of pressure applied to electrospun-film-based piezocapacitive sensors. The inset presents the pressure range from 0 to 0.4 kPa and shows the sensitivities of the sensors. **b** Schematic illustration of the working mechanism of the EMPA-based piezocapacitive sensor. **c** Transfer charge densities of electrospun-film-based TENGs under an impact force of 5 N. The inset presents a digital clock driven by the 3D EMPA-M-based TENG after clicking a mouse. **d–g** Superior performance of EMPA-based on-skin devices in health and finger manipulation monitoring in natural states. **d** Pictures showing the health monitoring for a driver. **e** Long-duration monitoring of the fingertip pulse waveform. The baseline fluctuation is associated with hand joint movement during the measurement. The insets show magnified fingertip pulse waveforms. **f** Pictures showing finger manipulation monitoring of clicking a mouse and three different states: (i) separation, (ii) light touch, and (iii) pressed state. **g** Synchronous current and relative capacitance change signals during finger manipulation monitoring of clicking a mouse. The insets show fingertip pulse waveforms in the separation state. The blue triangles represent the misidentification points due to the long response time of the piezocapacitive sensor.

In terms of the piezocapacitive sensors based on 3D EMPA-L, 3D EMPA-M, 3D EMPA-S, and 2D EP, the EMPAs significantly increase the sensitivity (i.e., the slope of the relative capacitance change–pressure curve), as shown in Fig. 5a and Supplementary Fig. 17b. This is because the gradient stress distribution of EMPAs makes the tips of micropyramids more compressed under applied pressure^16,45,58^, resulting in larger changes in the contact area between the electrode and the dielectric layer compared to the 2D EP (Fig. 5b). The sensor with the largest APH shows the highest sensitivity of 19 kPa^−1^ in the low-pressure range (≤200 Pa). In contrast, in the pressure range greater than 200 Pa, the highest sensitivity is found for the sensor with a medium APH of 18.23 μm (Supplementary Fig. 17c). This reversal probably results from the more suitable pyramid size of the 3D EMPA-M, which makes the contact area change larger under high pressure compared with 3D EMPA-L^59^. In addition, the 3D EMPA-M-based piezocapacitive sensor has an ultralow detection limit of 0.05 Pa, which enables detection of the weight of a mosquito by the sensor (Supplementary Fig. 17d). The sensor presents a response time of 48 ms for the maximum capacitance change of 80% (Supplementary Fig. 17e). Additionally, the sensor shows good durability for 1000 cycles of pressing at 2.3 kPa without performance degeneration (Supplementary Fig. 17f).

The EMPAs are also beneficial for triboelectric and piezoelectric performance. For the TENG, under a given impact force, the large contact area arising from the gradient space filling of EMPAs (Supplementary Fig. 18b) results in a high surface charge density and therefore boosted output^60,61^. Figure 5c and Supplementary Fig. 18e, f show the transfer charge densities, output voltages, and short-circuit current densities of TENGs based on depolarized 3D EMPA-L, 3D EMPA-M, 3D EMPA-S, and 2D EP. The EMPA TENGs have higher electrical outputs than the 2D electrospun-plane-based TENG. The highest electrical outputs are detected for the 3D EMPA-M-based TENG. This is also associated with the most suitable pyramid size of the 3D EMPA, which leads to the largest contact area^62^. Additionally, the 3D EMPA-M-based TENG attached to a fingertip can harvest biomechanical energy during clicking a mouse to drive a digital clock (inset in Fig. 5c and Supplementary Fig. 18g, h). To further explore the potential of the EMPA in high-performance TENGs, a flat electrospun PA66 film with high triboelectric positive polarity and the 3D EMPA-M are used as a pair of tribolayers (Supplementary Fig. 18i). The current density, voltage, transfer charge density, and energy conversion efficiency of the improved TENG are as high as 51.74 mA m^−2^, 1300 V, 105.1 μC m^−2^, and 42%, respectively (Supplementary Fig. 18j–l and Supplementary Note 8). In terms of PENGs, the gradient stress distribution of the EMPAs brings about stronger variations in the strain along the sample thickness direction^9,25^ and consequently higher piezoelectric outputs compared with the flat electrospun film. The piezoelectric current densities and voltages of the in situ polarized electrospun PVDF films are in line with the APH (Supplementary Fig. 21d–g). The improved electrical outputs of the TENGs and PENGs are emblematic of enhanced biomechanical energy harvesting and action sensing abilities.

The high sensitivity, ultralow detection limit and good imperceptibility make the EMPA-based piezocapacitive sensor suitable for long-duration bio-health monitoring for particular types of workers such as drivers without affecting their normal manipulation. Figure 5d, e shows the ultraweak fingertip pulses of a driver detected by the on-skin pressure sensor. After long-duration detection (> 4 h), the pulse signals do not show any degradation. Notably, the whole pulse waveform comprising a systolic peak (P_1_), a reflected systolic peak (P_2_), a dicrotic notch (P_3_), a diastolic peak (P_4_), and an end-diastolic notch (P_5_) is successfully recorded, indicating that the EMPA-based piezocapacitive sensor is highly sensitive. In contrast, the commercial photoplethysmograph loses the P_2_ peak that is important for estimating some physiological indexes, for example, the radial artery augmentation index^26,63^. Additionally, this on-skin pressure sensor avoids a series of problems arising from the commercial photoplethysmograph during long-term use, such as ischemic pressure necrosis, mechanical injury, and nail polish interference (Supplementary Note 9)^63^. Therefore, the EMPA pressure sensor is more acute and convenient for fingertip pulse detection and health diagnosis.

Furthermore, monitoring of both natural finger manipulation over a wide frequency range and mental or emotional conditions in real time is accomplished by using a hybrid sensor consisting of EMPA-based piezocapacitive (hereafter termed Sensor I) and triboelectric (Supplementary Fig. 23, hereafter termed Sensor II) sensors. This benefits from two facts: (i) The relative capacitance change arising from a fingertip pulse and hand joint movement is extremely small (*△C*/*C*_0_ ≤ 0.01, Fig. 5e), which indicates that fingertip pulse detection does not interfere with pressure sensing in manipulating objects. (ii) The ultrafast response (≤ 0.8 ms, Supplementary Fig. 24c) of the triboelectric sensor compensates for the drawback of the relatively slow response (48 ms) of the piezocapacitive sensor.

Given the requirement of interference-free multiple information monitoring for eSports players^64^, the hybrid sensor is suitable for these players to score higher. The synchronous signals from this sensor can be used to distinguish complicated manipulation details of an eSports player, with Sensor I and Sensor II attached to a fingertip and a mouse, respectively (Fig. 5f). As shown by the green dotted line in Fig. 5g, the force (*F*) threshold for the mouse to be triggered is 0.8 *N*. Three different states of separation (*F* = 0 *N*, Fig. 5f-i), light touch (0 *N* < *F* ≤ 0.8 *N*, Fig. 5f-ii), and pressure (*F* > 0.8 *N*, Fig. 5f-iii) can be identified by the force-dependent relative capacitance change curve (red curve in Fig. 5g). Accordingly, single click (yellow region in Fig. 5g), multiple clicks (pale orange region in Fig. 5g), long press (purple region in Fig. 5g), and invalid click (pink region in Fig. 5g) are further recognized based on the time span. In terms of very quick multiple clicks (orange region in Fig. 5g), misidentification points are observed from Sensor I (blue triangles in Fig. 5g and Supplementary Fig. 24a, b). The ultrafast response and insensitivity in the pressed state (which is contrary to the piezocapacitive sensor, Supplementary Fig. 24d–f) of Sensor II help accurately detect instantaneous contact and separation (navy blue curve in Fig. 5g). Note that the introduction of a triboelectric current for sensing is ascribed to both the triboelectric open-circuit voltage and transfer charge signals featuring uneven baselines (Supplementary Fig. 24f–h).

Notably, the hybrid sensor manifests superb stability in fingertip pulse detection during finger manipulation monitoring. As shown in the insets in Fig. 5g, in the separation state, Sensor I continuously records fingertip pulses and presents stability after 550 clicks and 2 heavy pressures of approximately 20 *N*. All this information can be used to calculate important competence evaluation indexes such as actions per minute. Additionally, it can provide mental stress and emotion information of the eSports player through the pulse rate (PR) and pulse rate variability (PRV, Supplementary Fig. 25)^64–66^.

## Discussion

In this work, we have developed versatile EMPAs that combine gradient micropyramid geometry with outstanding multidisciplinary properties and ultrathin, ultralight, gas-permeable structure to endows various on-skin devices with both superior performance and good imperceptibility by the unique electrospinning self-assembly. During the self-assembly process, wet heterostructured electrified jets made from various materials (including PVDF, TPU, and PVA) accumulate into structurally designable EMPAs (with unit size from micron to millimeter scale). We systematically investigated the growth process and various structural control approaches of EMPAs to make on-skin devices competent for application in daytime radiative cooling, pressure sensing, and bioenergy harvesting. Compared to other similar devices, the EMPA devices show competitive performances (Supplementary Tables 4–6). The EMPA-based radiative cooling fabric (as thin as 47 µm) with high vis-NIR reflectivity (97.9%) and MIR emissivity (76.3%) reduces the temperature by ~4 °C under a solar intensity of 1 kW m^−^^2^ and provides long-term comfort. Furthermore, the high sensitivity (19 kPa^−^^1^), ultralow detection limit (0.05 Pa), and ultrafast response (≤0.8 ms) of the EMPA piezocapacitive-triboelectric hybrid sensor enable acute detection of ultraweak fingertip pulses during monitoring of natural finger manipulation to analyze health conditions and manipulation details simultaneously. In addition, the boosted triboelectric and piezoelectric outputs (105.1 μC m^−^^2^) of EMPA nanogenerators facilitate reliable biomechanical energy harvesting. Notably, the performances and applications could be further improved and broadened, respectively, by optimizing the materials and structures of EMPAs. This work paves the way for assembling such versatile EMPAs and opens up many opportunities for the application of on-skin devices in effective individual protection and healthcare, sensitive sensing, high-power self-powered electronics, and human-machine interaction with minimal sensory interference.

## Methods

### Fabrication of electrospun films and on-skin devices

All electrospun films including pyramid-arrayed and flat films were prepared using an electrospinning machine (DP30, Tianjin Yunfan Instrument Co., Ltd., China). The as-prepared electrospun films were directly used as radiative cooling electrospun fabrics. Both the electrospun-film-based piezocapacitive sensor and TENG consisted of two Au-coated electrospun films. The conventional micropyramid-arrayed piezocapacitive sensor consisted of Au-coated polypropylene and PDMS films. The electrospun film sandwiched between two Au electrodes formed the PENG. The detailed information regarding materials, precursor solutions, electrospinning parameters, and fabrication process of various on-skin devices can be found in Supplementary Methods and Supplementary Table 1 and 2.

### Characterization

The microscopic architectures and crystalline structures of electrospun films were characterized by field-emission scanning electron microscopy (GeminiSEM 500, Zeiss, German), laser confocal microscope (OLS5100, Olympus, Japan and LSM 800, Zeiss, German), differential scanning calorimetry (DSC Q2000, TA Instruments, America), and Fourier transform infrared spectroscopy (Tensor II, Bruker, Germany). The substrate thickness, pyramid vertex interval, pyramid height, and percentage of surface fibers were measured using the software of Adobe Photoshop 2021, Nano Measurer 1.2, and Zen 2.6 system. A digit multimeter (DMM7510, Tektronix, America) was used to test the resistance. The surface potential was recorded by an electrostatic voltmeter (JH-TEST, Zejing, Japan). The local electric field simulation was performed by COMSOL Multiphysics 6.0 and SolidWorks 2016 software (see details in Supplementary Methods). The breathability was evaluated by water weight loss method (see details in Supplementary Methods). A multi-dimensional force sensor (Shenzhen Ruilide Technology Co., Ltd., China) was used to record the simultaneous load force and grip force. The visible to near-infrared reflectivity of the electrospun film was determined by a UV-VIS-NIR spectrophotometer (UV-3600, Shimadzu, Japan). For the mid-infrared emissivity spectra, a Fourier transform infrared spectrometer (Nicolet iS50, Thermo Scientific, America) was used. Change in capacitance of the piezocapacitive sensor was determined by a LCR meter (E4980A, Agilent, America) and an impedance analyzer (IM 3570, Hioki, Japan). A high-precision mechanical system (VS-15-4, Newmark Systems, America) was used to control the pressure applied to the sensor. A custom-made cycle impact apparatus with a step motor linked to a slider by means of a connecting rod was used to exert given pressure on sensors and nanogenerators at a given frequency (Supplementary Fig. 18d, 21c, and 24a). The applied force was recorded by two types of digital force gauges (M5-05 and M5-5, Mark-10, America). The patting force was detected by a S-shaped digital force gauge (DS2-200N-S, Zhiqu, China). An oscilloscope (MSO44, Tektronix, America) and an electrometer (6514, Keithley, America) were used to detect the electrical outputs of nanogenerators. Radiative cooling performance was tested using an infrared thermal imaging camera (E6, FLIR, America) and Pt100 resistance temperature detectors interfaced with a multi-channel datalogger (MIK-R9600, Meacon, China). A Xenon lamp (CEL-S500/350, Ceaulight, China) and an optical power densitometer (CEL-NP2000, Ceaulight, China) were used to simulate solar radiation with a power of 1 kW m^−^^2^. The average temperature was calculated by the software of FLIR Tools according to the thermal camera image. The average values of the temperature drop, piezocapacitive sensing sensitivity, and electrical output were calculated according to five identical devices for each type of electrospun film.

### Human research participants

Twenty adults aged 21–31 participated in the biocompatibility study, object-grasping experiment, and fingertip pulse waveform long-duration monitoring. Relevant details are illustrated in Supplementary Methods and Supplementary Table 3. The purposes and significances of the survey and the experiment were informed to the participant before the survey. Participants provided informed consent prior to the experiment. We promised that all information just would be used for research and kept strictly confidential. Meanwhile, the experiment was approved by Shanxi Provincial People’s Hospital Research Ethics Committee (approval number: 2021-323).

## Supplementary information

Supplementary Infomation

## Data Availability

The data generated in this study are provided as a Source Data file. They are also available from the corresponding author upon reasonable request. Source data are provided with this paper.

## Source data

Source Data
