# Supplementary material for: Versatile self-assembled electrospun micropyramid arrays for high-performance on-skin devices with minimal sensory interference
Source: Nat Commun. 2022 Oct 3;13:5839. doi: 10.1038/s41467-022-33454-y (PMC9530173; doi:10.1038/s41467-022-33454-y)
Supplement: Supplementary file 1 — Supplementary Infomation [file 41467_2022_33454_MOESM1_ESM.pdf]

Supplementary Information

Versatile Self-Assembled Electrospun Micropyramid Arrays for High-Performance On-Skin Devices with Minimal Sensory Interference

Jia-Han Zhang[1], Zhengtong Li[2], Juan Xu[3], Jiean Li[1], Ke Yan[1], Wen Cheng[1], Ming Xin[1], Tangsong Zhu[4], Jinhua Du[5,6], Sixuan Chen[7], Xiaoming An[4], Zhou Zhou[1], Luyao Cheng[1], Shu Ying[1], Jing Zhang[1], Xingxun Gao[1], Qiuhong Zhang[4], Xudong Jia[4], Yi Shi[1]* & Lijia Pan[1]*

[1]Collaborative Innovation Center of Advanced Microstructures, School of Electronic Science and Engineering, Nanjing University, Nanjing 210093, China

[2]Key Laboratory of Hydrology Water Resources and Hydraulic Engineering, Hohai University, Nanjing 210098, China

[3]Shanxi Provincial People's Hospital, Taiyuan 030012, China

[4]School of Chemistry and Chemical Engineering, Nanjing University, Nanjing 210093, China

[5]School of Materials and Metallurgy, Inner Mongolia University of Science and Technology, Baotou 014010, China

[6]School of Chemistry and Chemical Engineering, Inner Mongolia University of Science and Technology, Baotou 014010, China

[7]School of Physics, Nanjing University, Nanjing 210093, China

Table of Contents

1. Supplementary Methods....S4
1.1 Fabrication of electrospun pyramid arrays and flat electrospun films....S4
1.2 Fabrication of radiative cooling electrospun fabrics....S4
1.3 Fabrication of electrospun-film-based piezocapacitive sensors....S5
1.4 Fabrication of on-skin piezocapacitive sensors....S5
1.5 Fabrication of electrospun-film-based TENGs....S5
1.6 Fabrication of electrospun-film-based PENGs....S6
1.7 Local electric field simulation....S6
1.8 Characterization of breathability....S7
1.9 Human research participants....S7
1.10 Schematic diagram drawing....S8

2. Supplementary Figures....S9
2.1. Electrospinning apparatus to fabricate electrospun pyramid structures....S9
2.2. Surface morphology of the aluminum foil....S10
2.3. Morphologies of various electrospun PVDF pyramid structures....S11
2.4. Formation process of differently charged initial fibers....S13
2.5. Surface potential before electrospinning....S14
2.6. Experimental procedure of resistance measurement....S15
2.7. Surface potential of the deposited EMPA film during electrospinning....S16
2.8. Surface fiber density of the electrospun micropyramid....S17
2.9. Electric field simulation during the self-assembly process....S18
2.10. Size tunability for self-assembly electrospinning of EMPAs....S19
2.11. Characteristics of three types of EMPA-based films and the flat electrospun film....S20
2.12. Shape of jets capable of composing EMPAs....S21
2.13. Morphologies of films fabricated via dry heterostructured electrified jets....S22
2.14. Gas permeability and biocompatibility measurements....S23
2.15. Slip ratio experiment....S24
2.16. Optical properties, test setup and radiative cooling performance of electrospun films....S25
2.17. Electrical characterization of EMPA-based piezocapacitive sensors....S27
2.18. Working mechanism, test setup and electrical performance of electrospun film-based TENGs....S29
2.19. Energy conversion efficiency test of TENGs....S31
2.20. Estimation of the biomechanical-to-electrical energy conversion efficiency....S32

2.21. Characterization of EMPA-based piezoelectrics and corresponding PENGs. S33
2.22. Fingertip pulse waveforms detected by different devices. S35
2.23. Working mechanism of EMPA-based triboelectric sensor attached to a mouse. S36
2.24. Electrical characterizations of triboelectric and piezocapacitive sensors. S37
2.25. PRV test. S39

3. Supplementary Notes. S40
3.1 Supplementary Note 1: Formation process of wet heterostructured jets. S40
3.2 Supplementary Note 2: Formation process of differently charged initial fibers. S41
3.3 Supplementary Note 3: Necessity of the wet heterostructured electrified jets for fabricating EMPAs. S44
3.4 Supplementary Note 4: The reason why negative charges appear on the tops of electrospun microdomes and micropyramids, and the reason why the local electric field is higher at a position closer to the tip of the microdome or micropyramid. S45
3.5 Supplementary Note 5: Dependence study between voltage and size of EMPAs. S46
3.6 Supplementary Note 6: Removal of the interaction between the finger and the object. S47
3.7 Supplementary Note 7: Purpose of the experiment about the EMPA films with different APHs. S48
3.8 Supplementary Note 8: Energy conversion efficiency of the improved TENG. S49
3.9 Supplementary Note 9: Drawbacks of the commercial photoplethysmograph. S51

4. Supplementary Tables. S52
4.1 Supplementary Table 1 Materials for fabricating various electrospun films. S52
4.2 Supplementary Table 2 Electrospinning parameters for fabricating various electrospun films. S53
4.3 Supplementary Table 3 VAS grading scale for biocompatibility study. S55
4.4 Supplementary Table 4 Cooling performance comparison among the EMPA films and other gas-permeable fabrics reported in recent years. S56
4.5 Supplementary Table 5 Pressure sensing performance comparison among EMPA-based devices and other similar devices with different microstructures. S57
4.6 Supplementary Table 6 Energy harvesting performance comparison among the EMPA-based nanogenerators and other all-fiber and nanofiber-based nanogenerators. S59

5. Supplementary References S61

1. Methods.

Supplementary Methods

Fabrication of electrospun pyramid arrays and flat electrospun films. The electrospinning solutions used in this work were fabricated by dissolving polymers (PVDF, TPU, and PVA) in solvents (dimethyl sulfoxide (DMSO), acetone, N,N-dimethylformamide, and water). The detailed material information and solution compositions are respectively presented in Supplementary Tab. 1 and 2. All resultant electrospun films were prepared using an electrospinning machine (DP30, Tianjin Yunfan Instrument Co., Ltd.). The electrospinning system consisted of a 5 mL syringe with the solution flow rate ranging from 0.5 to 1.0 mL h^{-1}, a 22-gauge spinneret connected to a power supply over the positive voltage range of 10.0–25.0 kV, and an electrically grounded drum collector with 20 rpm rotating speed (Supplementary Fig. 1). A programmable dehumidifier (CS002, Wuzhao Power Technology Co., Ltd., China) and heater were respectively used to control the humidity and temperature during electrospinning. A smooth aluminum foil was wrapped around the drum to collect electrospun fibers. The arithmetical mean height, maximum peak height, and maximum pit height of the aluminum foil are 21.9, 208, and 209 nm, respectively. After electrospinning, as-prepared samples were dried at room temperature until solvent completely volatilized to obtain easy-to-release electrospun films containing 3D micropyramid arrays or 2D planes. More detailed electrospinning parameters for fabricating various electrospun films are presented in Supplementary Tab. 2.

Fabrication of radiative cooling electrospun fabrics. Four electrospun PVDF films, 3D EMPA-L, 3D EMPA-S, 3D EMPA-M, and 2D EP, were directly used as radiative cooling electrospun fabrics (Fig. 4d). The micropyramid-arrayed side of the electrospun PVDF film faced solar irradiation.

Fabrication of electrospun-film-based piezocapacitive sensors. The electrospun-film-based piezocapacitive sensor was composed of two layers, a top Au flat electrospun film and a top Au electrospun micropyramid-arrayed film, which clung to each other (Supplementary Fig. 17a). For comparison, the reference sample comprised two top Au flat electrospun films. The Au was deposited using magnetron sputtering (K550X, Emitech, England). The working area for all piezocapacitive sensors was 10×10 mm^2. The piezocapacitive sensor was stuck to a glass substrate and covered by a smooth protective layer for the sake of relative capacitance change versus pressure curve test.

Fabrication of on-skin piezocapacitive sensors. In terms of EMPA-based piezocapacitive sensor, the PVA nanofibers were deposited onto a fingertip by electrospinning and were then wetted by water mist to firmly stick the sensor to the fingertip (Fig. 3c and Supplementary Fig. 14f). The non-working area between the flat electrospun film and the EMPAs was also treated through wet electrospun PVA nanofibers to guarantee fine contact between two layers of the sensor. Body temperature can soon dry out gas-permeable PVA glue within 3 min. Subsequently, the on-skin EMPA-based sensor started to record fingertip pulses and pressure signals (Fig. 5d, f). Similarly, a top Au flat polypropylene film and a top Au micropyramid-arrayed PDMS film made up the conventional PDMS micropyramid-array-based piezocapacitive sensor (Supplementary Fig. 14e)[1]. Due to the unsatisfactory flexibility, large thickness and heavy weight of this conventional sensor, ordinary glues cannot stick it to the finger. Therefore, this conventional sensor was fixed on the fingertip by wrapping a scotch tape (Supplementary Fig. 14e). The working area of the two types of aforementioned on-skin sensors was approximately 10×10 mm^2.

Fabrication of electrospun-film-based TENGs. Three types of TENGs were used in this work. The first comprised a top Au flat electrospun film and a top Au electrospun micropyramid-arrayed film with a maximum gap of 25 mm between these two films (Supplementary Fig. 18a, b, d). For comparison, the reference sample was comprised of two

top Au flat electrospun films. Performance comparison of EMPAs with different micropyramid sizes was investigated through this type of TENG with a working area of $20 \times 20\ mm^2$. The second with a working area of $45 \times 45\ mm^2$ included a top Au flat electrospun poly(hexamethylene adipamide) (PA66) film and a bottom Au electrospun micropyramid-arrayed PVDF film (Supplementary Fig. 18i), which served as a high-efficiency mechanical energy harvester. The third with a working area of $15 \times 15\ mm^2$ included a top Au flat electrospun PA66 film and a bottom Au electrospun micropyramid-arrayed PVDF film (Supplementary Fig. 23 and Fig. 5f). These two electrospun films (i.e., a pair of tribolayers) are in contact to monitor contact and separation states during clicking mouse. In the biomechanical energy harvesting experiment, the working area of the TENG consisting of a top Au 3D EMPA-M and a top Au 2D EP was $10 \times 10\ mm^2$ (Supplementary Fig. 18b). It is noteworthy that all PVDF samples were depolarized by annealing at 150 °C for 24 h to avoid interference from piezoelectric effect[2] and the annealing procedure exerted no influence on sample morphology (Supplementary Fig. 18c).

Fabrication of electrospun-film-based PENGs. The electrospun piezoelectric PVDF film was sandwiched between two Au electrodes, and then the resultant device was encapsulated using PI tapes. Meanwhile, the test surface of the cycle impact apparatus was also covered by a piece of PI film to minimize the interference from triboelectric charges[3] (Supplementary Fig. 21c). The working area for all PENGs was $20 \times 20\ mm^2$.

Local electric field simulation. All models were built by SolidWorks 2016 software. The model of the electrospinning machine consisted of a box full of air ($650 \times 600 \times 450\ mm^3$), a grounded aluminum collector (length 300 mm), and a steel spinneret (external diameter 0.71 mm, internal diameter 0.41 mm, length 28 mm). The distance from the spinneret to the collector was 80 mm. The applied voltage was +12.5 kV. The models of the electrospun microdome and micropyramid were 5.12 and 26.1 μm in height, respectively. Subsequently, the models were imported into COMSOL Multiphysics 6.0 with an AC/DC module for

electrostatic field simulation. Preference was given to the software material library: air (Air), aluminum (UNS A91050), and steel (UNS S31600).

Characterization of breathability. Deionized water of 1 g was placed in a glass bottle with a height of 98 mm and a diameter of 15 mm at the opening, and then the sample was attached to the opening of the bottle (Supplementary Fig. 14a). Subsequently, a glass cover was used to avoid the interference from strong winds in the constant climate chamber (Supplementary Fig. 14b). Additionally, there was a 32 mm gap between the glass cover and the turntable, which made the temperature and humidity inside and outside the glass cover consistent. The turntable with a rotation speed of 3 rpm was used to make any possible interference towards each of the test samples equal (Supplementary Fig. 14b). Finally, the installation was stored in the constant climate chamber at 25 °C and a humidity of 30% (Supplementary Fig. 14c). Meanwhile, the subsequent decrease in weight was tested.

Human research participants. Twenty adults aged 21–31 participated in the biocompatibility study, object-grasping experiment, and fingertip pulse waveform long-duration monitoring.

Biocompatibility study and fingertip pulse waveform long-duration monitoring. Twenty adults aged 21–31 took part in this study. The electrospun and conventional PDMS micropyramid-array-based piezocapacitive sensors were attached to fingertips for 7 h. Participants reported any feelings during the duration of the tests for each of the two sensors. The VAS (0–10) was used for this report, and the details are illustrated in Supplementary Tab. 3. One healthy male aged 27 took part in the fingertip pulse waveform long-duration monitoring. The questionnaire survey was conducted anonymously.

Object-grasping experiment. Eighteen right-handed participants aged 21–30 with no reported neurological disorders took part in this measurement. Participants were seated in front of an instrumented object which rested on an opaque box to avoid the visual influence. A multi-dimensional force sensor (Shenzhen Ruilide Technology Co., Ltd., China) embedded in the

object was used to record the simultaneous load force and grip force between the thumb and index fingers. The sensor-equipped object weighed 58 g (corresponding to a load force of 0.57 N). Different loads (50, 100 or 150 g) could then be suspended from the bottom of the object to change the load force (1.06, 1.55 or 2.04 N). The recorded load force of the object was programmatically corrected before the test. The monitor rendered acoustic control of the timing of the task. Each participant lifted the object 3 cm above the box and hold it for 5 s by thumb and index fingers. Subsequently, the object was placed down. Three finger surface conditions (bare finger, EMPA device, and conventional PDMS-based device) were randomly ordered across participants. Within each condition, lifts with the four different load forces were conducted in a randomized order, with each lift performed 10 times with the same load force.

To remove the effect of the interaction between the finger and the object on the grip force, a slip ratio experiment was carried out. After the object was lifted up, participants were asked to slowly release the force of the fingers until the object drops. The last grip force prior to the object slipping from the grasp was used to estimate the minimum level of the grip force for every load force (Supplementary Fig. 15a). The median values over five trials for each participant are adopted. The relationship among the minimum grip force ($F_{g'}$), the load force (F_l), and the friction coefficient (μ_f) meets the following equation:

$$F_l = 2\, F_{g'}\, \mu_f \tag{1}$$

The additional grip force was obtained by subtracting the minimum grip force based on the grasp friction coefficient (Supplementary Fig. 15b) from the actually used grip force. Finally, p-values of post hoc tests were calculated based on two-factor ANOVA without replication.

Schematic illustration drawing. The first author, J.-H. Zhang, used SolidWorks 2016, 3ds Max 2020, PhotoView 360 and V-Ray 5.0 rendering software to draw all the 3D schematic illustrations. He used a digital art sketch tablet (G22, Gaomon, China) with Adobe Photoshop 2021 and Adobe Illustrator 2021 software to draw all the 2D schematic illustrations.

2. Supplementary Figures.

2.1. Electrospinning apparatus to fabricate electrospun pyramid structures.

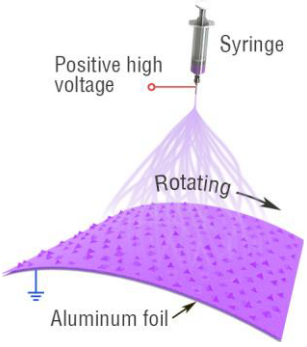

Supplementary Figure 1 Electrospinning apparatus to fabricate electrospun pyramid structures. Schematic diagram of the electrospinning apparatus.

2.2. Surface morphology of the aluminum foil.

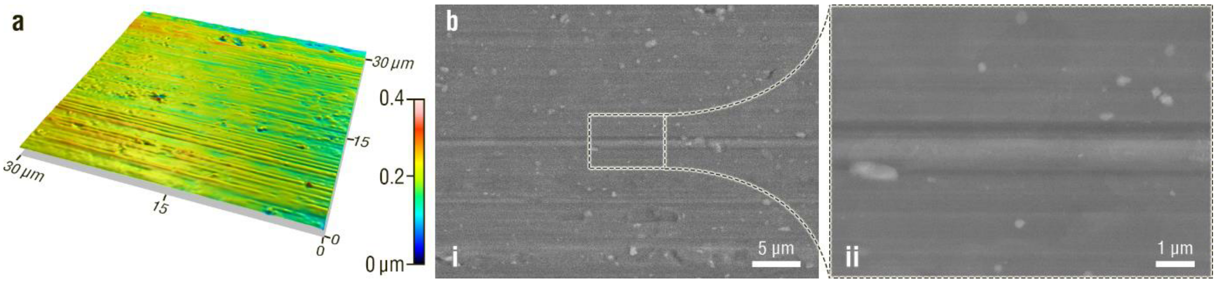

Supplementary Figure 2 Surface morphology of the aluminum foil. a Laser confocal microscopy image of the aluminum foil. **b** (i) SEM image and (ii) a corresponding magnified SEM image of the aluminum foil. The arithmetical mean height, maximum peak height, and maximum pit height of the aluminum foil are 21.9, 208, and 209 nm, respectively. The aluminum foil surface consists of lots of stripe-like structures, some dome-like protuberances, and a small number of pits. There exists a great difference between the surface morphology of the aluminum foil and the electrospun micropyramid array, avoiding the possibility that the formation of EMPAs originates from the microstructures of the aluminum foil.

2.3. Morphologies of various electrospun PVDF pyramid structures.

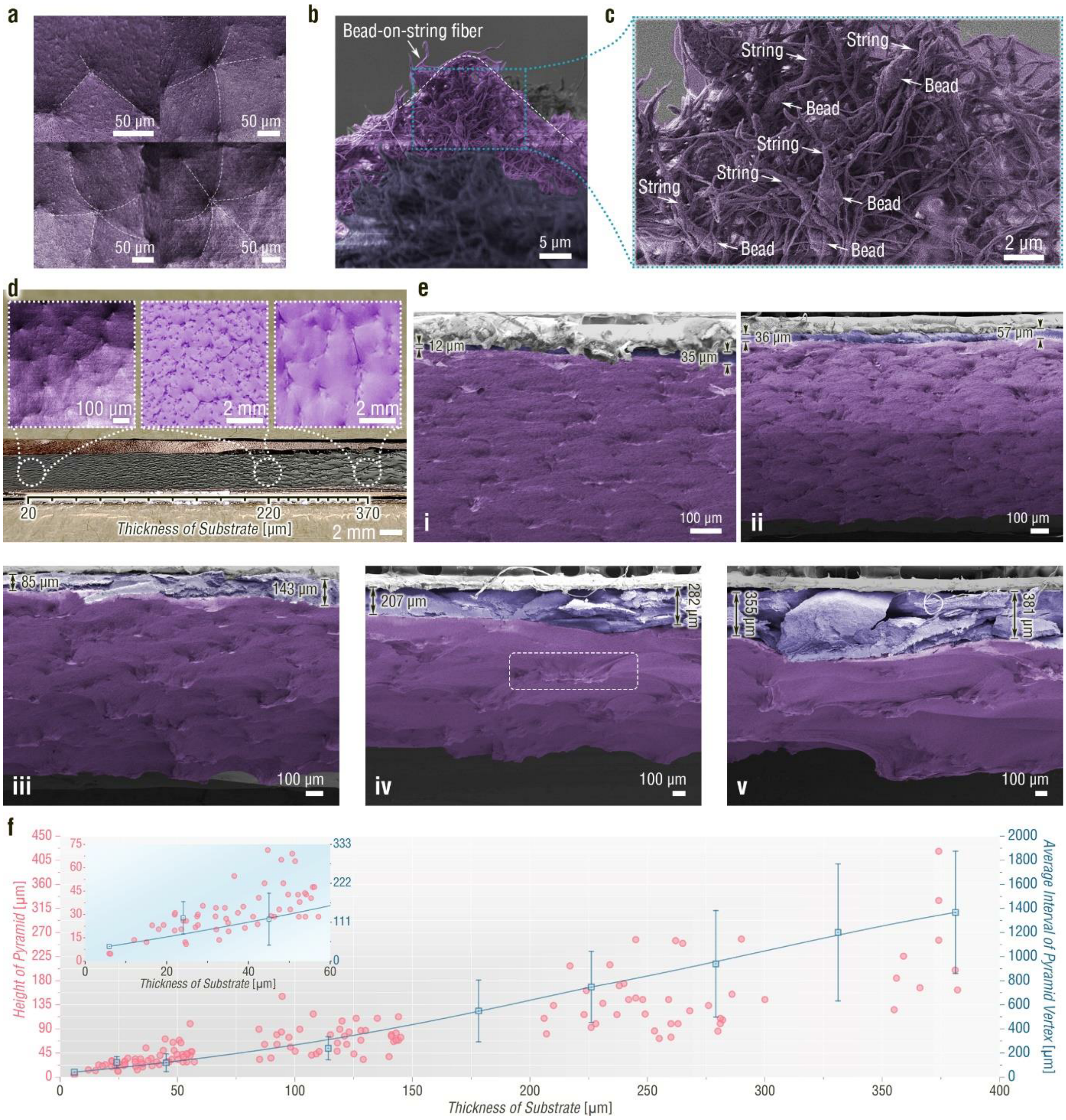

Supplementary Figure 3 Morphologies of various electrospun PVDF pyramid structures. **a** SEM images showing the arris number of the electrospun micropyramid structure. **b** Cross-sectional SEM image of a dissected electrospun micropyramid. **c** Magnified SEM image of the dissected electrospun micropyramid shown in Supplementary Fig. 3b. **d** Photograph showing gradually thickened electrospun film with growing size of electrospun pyramid arrays. The electrospun film was blackened by Au sputtering to present EMPAs more clearly. The insets show various electrospun pyramid arrays with different substrate thicknesses. **e**

SEM images of electrospun pyramid arrays with the gradually thickened electrospun substrate. The section included in the dashed box showing multiple vertexes existing in a single 3D protuberance is an intermediate state of fusion. **f** Dependence of height of pyramid, average interval of pyramid vertex and thickness of substrate. The inset shows the enlarged view of height of pyramid and average interval of pyramid vertex over the substrate thickness within 60 μm. Error bars represent standard deviation.

2.4. Formation process of differently charged initial fibers.

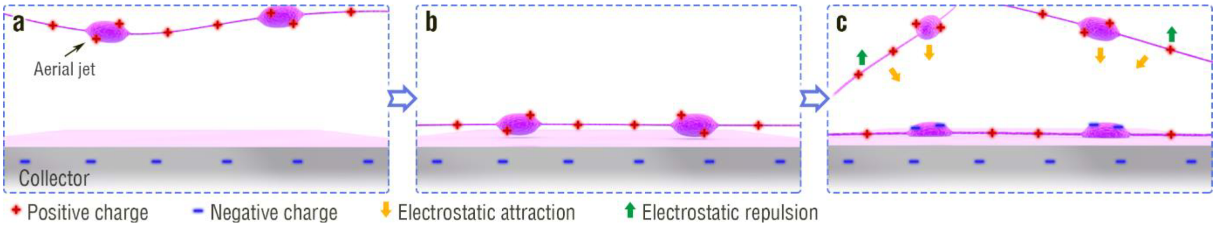

Supplementary Figure 4 Formation process of differently charged initial fibers. Schematic illustrations of **a** positively charged aerial jet, **b** positively charged aerial jet close to the collector, and **c** differently charged initial fiber. The schematic illustrations visualize a probable state of the differently charged initial fibers.

2.5. Surface potential before electrospinning.

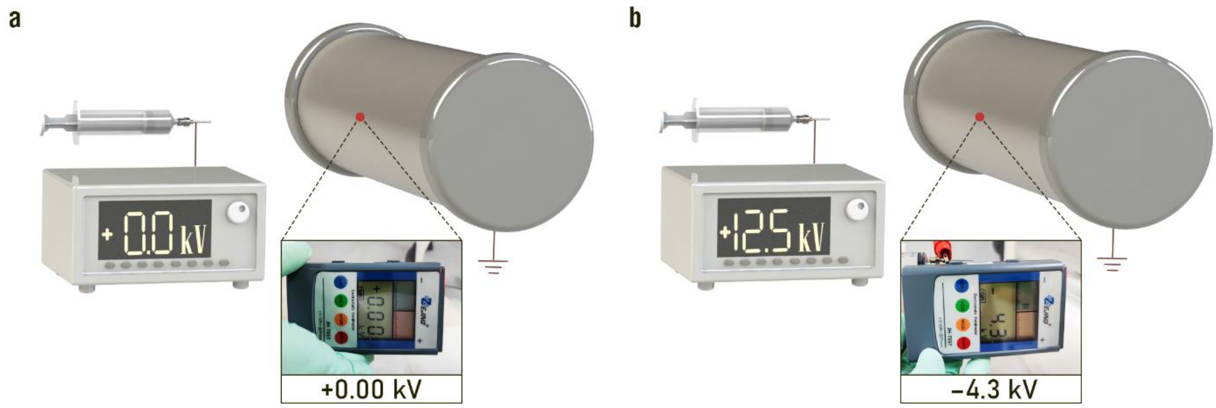

Supplementary Figure 5 Surface potential before electrospinning. a Surface potential of the aluminum foil at the electrospinning voltage of 0 kV. **b** Surface potential of the aluminum foil at the electrospinning voltage of +12.5 kV.

2.6. Experimental procedure of resistance measurement.

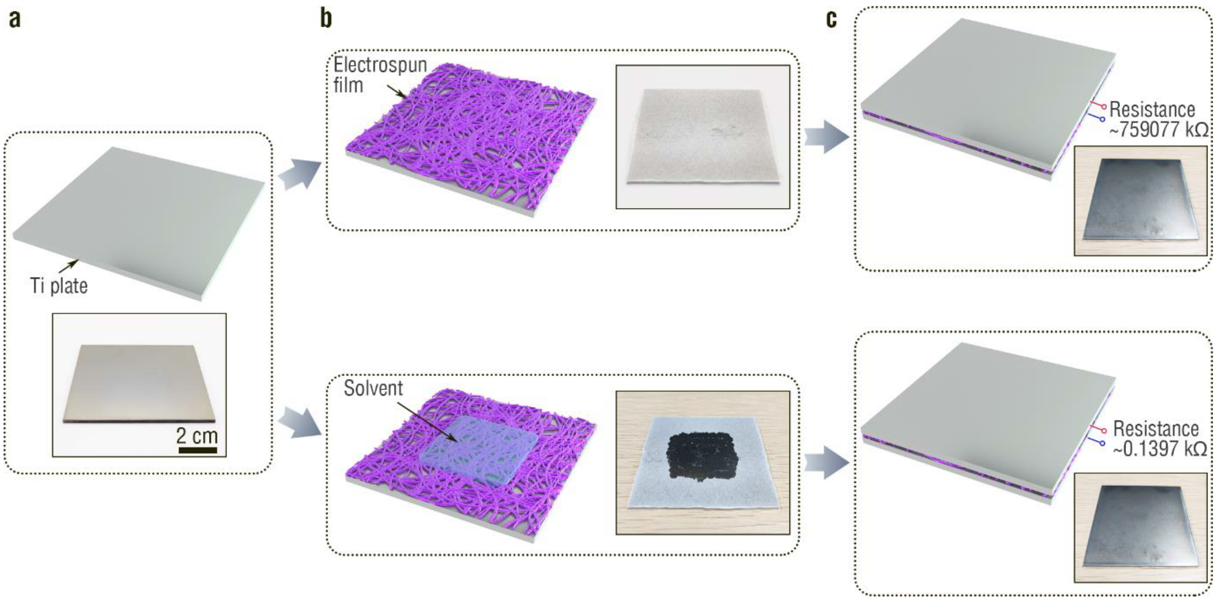

Supplementary Figure 6 Experimental procedure of resistance measurement. a Schematic diagram and photograph of the Ti plate. **b** Schematic diagrams and photographs showing an electrospun film deposited on the Ti plate and an electrospun-film-deposited plate wetted with solvent. **c** Schematic diagrams and photographs showing the dry electrospun film and the wet electrospun film sandwiched between two Ti plates, respectively.

2.7. Surface potential of the deposited EMPA film during electrospinning.

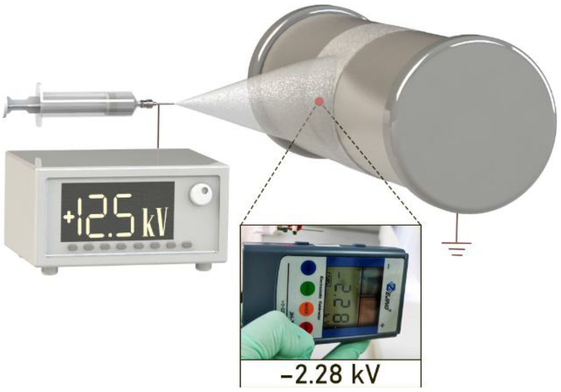

Supplementary Figure 7 Surface potential of the deposited EMPA film during electrospinning. Surface potential of the deposited EMPA PVDF film during electrospinning.

2.8. Surface fiber density of the electrospun micropyramid.

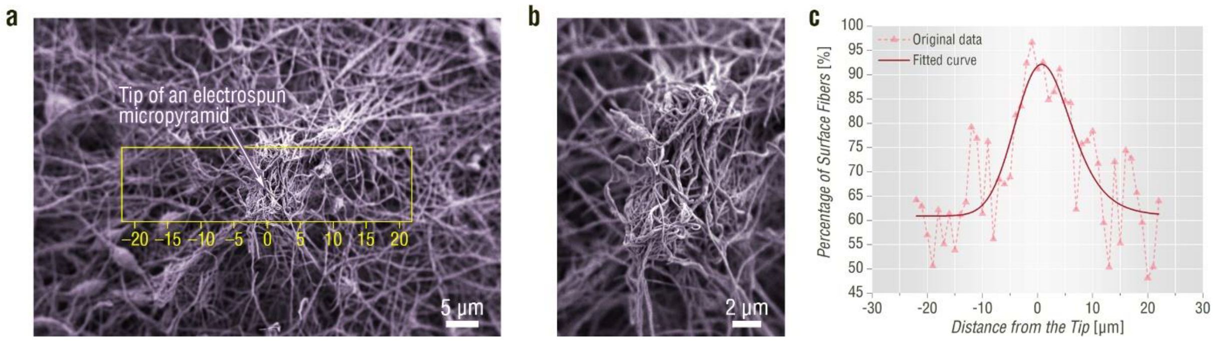

Supplementary Figure 8 Surface fiber density of the electrospun micropyramid. a SEM image of an electrospun micropyramid. **b** Magnified SEM image showing the tip of an electrospun micropyramid. **c** Percentage of surface fiber as a function of the distance from the tip of the electrospun micropyramid. The SEM image information in the yellow box is transformed into quantitative data in Supplementary Fig. 8c. The fitted curve is based on GaussMod.

2.9. Electric field simulation during the self-assembly process.

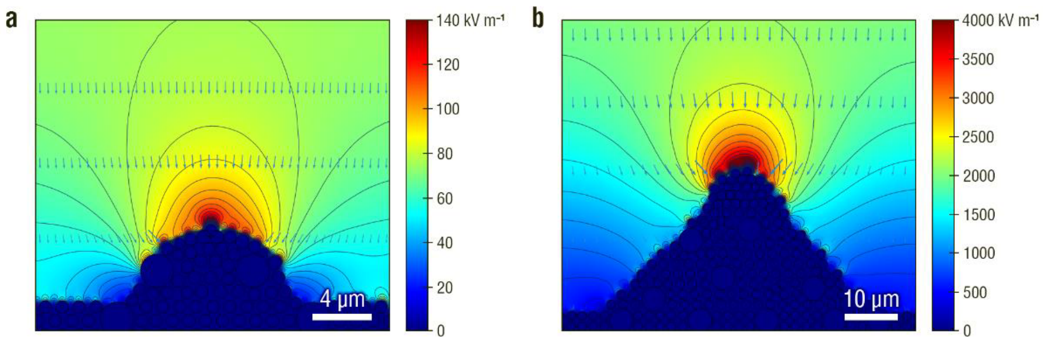

Supplementary Figure 9 Electric field simulation during the self-assembly process. a Computational electric field distortion and intensity near an electrospun microdome. **b** Computational electric field distortion and intensity near an electrospun micropyramid.

2.10. Size tunability for self-assembly electrospinning of EMPAs.

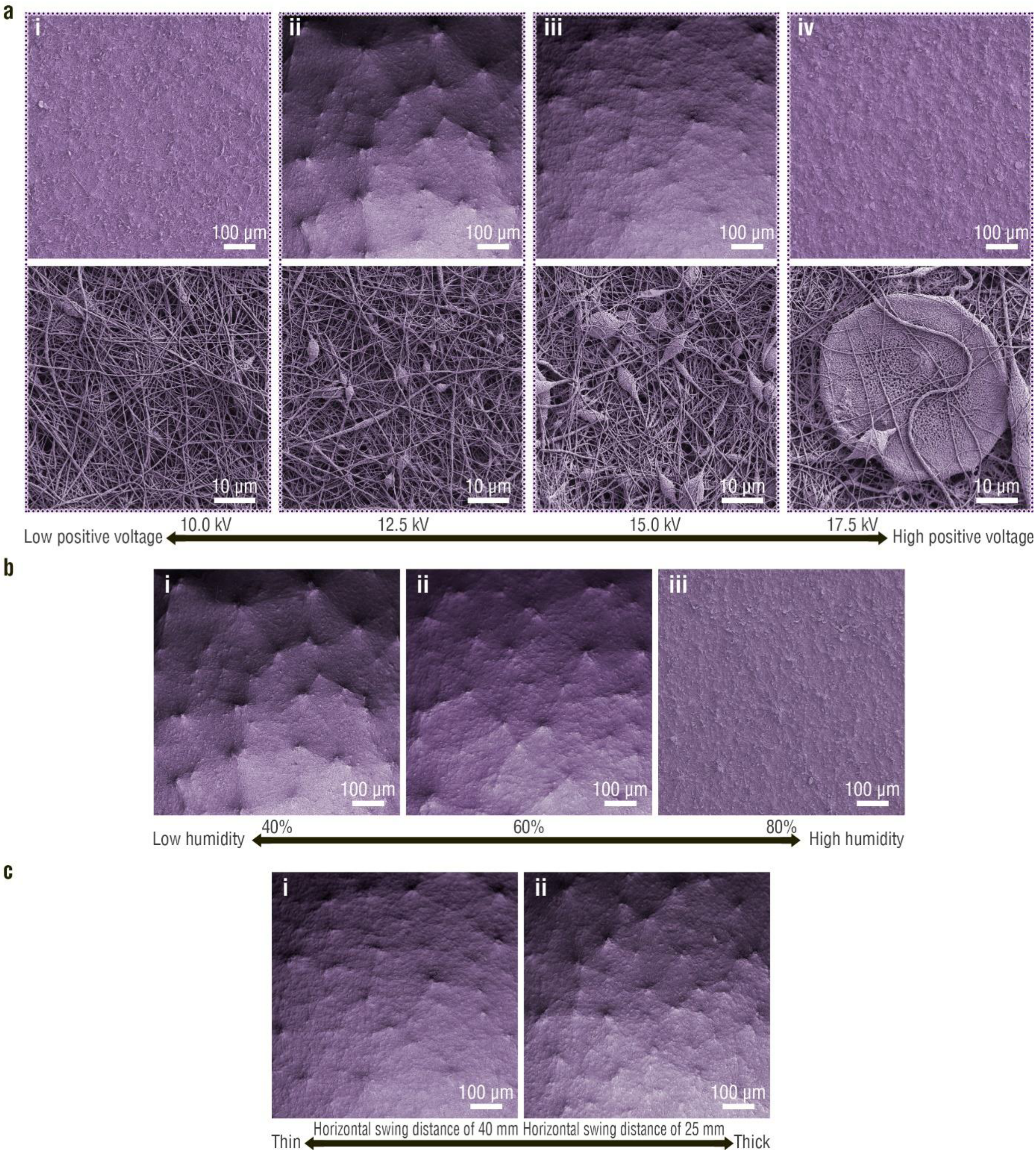

Supplementary Figure 10 Size tunability for self-assembly electrospinning of EMPAs. a SEM images of electrospun fibers fabricated at the positive voltage of (i) 10.0, (ii) 12.5, (iii) 15.0, and (iv) 17.5 kV. **b** SEM images of electrospun films fabricated at the relative humidities of (i) 40%, (ii) 60%, and (iii) 80%. **c** SEM images of electrospun films fabricated at the horizontal swing distances of (i) 40 and (ii) 25 mm.

2.11. Characteristics of three types of EMPA-based films and the flat electrospun film.

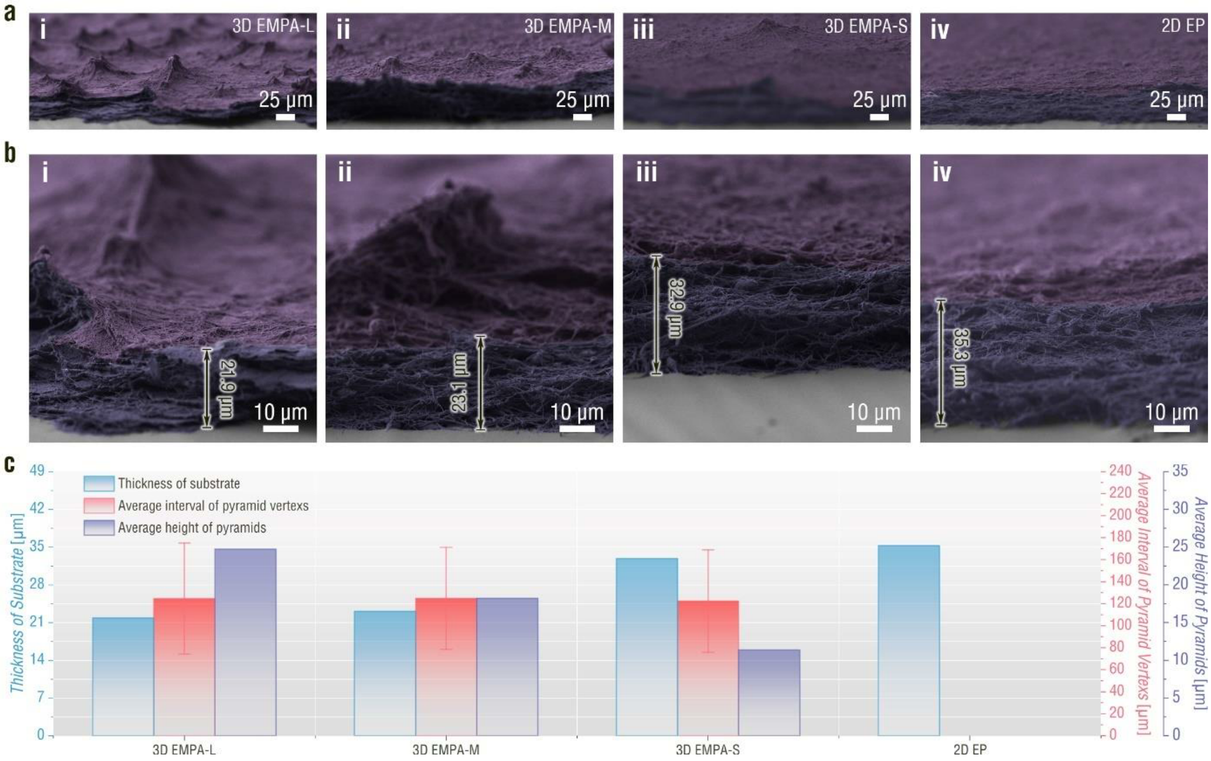

Supplementary Figure 11 Characteristics of three types of EMPA-based films and the flat electrospun film. a Cross-sectional SEM images of the pyramidal-microstructured electrospun films with the average pyramid heights of (i) 24.75, (ii) 18.23, and (iii) 11.37 μm and (iv) the flat electrospun film. **b** SEM images showing the thicknesses of (i) 3D EMPA-L, (ii) 3D EMPA-M, (iii) 3D EMPA-S, and (iv) 2D EP. **c** Substrate thicknesses, average pyramid vertex intervals, and average pyramid heights for 3D EMPA-L, 3D EMPA-M, 3D EMPA-S, and 2D EP. Error bars represent standard deviation.

2.12. Shape of jets capable of composing EMPAs.

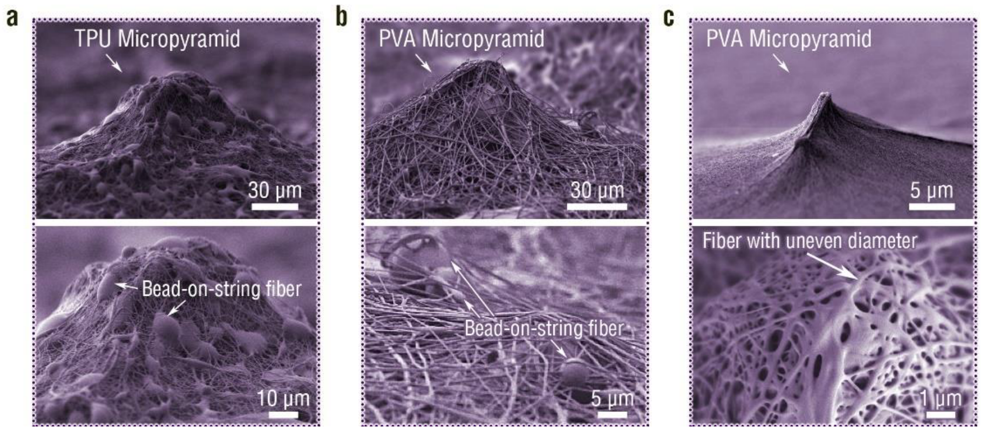

Supplementary Figure 12 Shape of jets capable of composing EMPAs. SEM images showing three kinds of heterostructured jets: (**a**, **b**) bead-on-string TPU and PVA jets, and (**c**) fibrous PVA jets with uneven diameter.

2.13. Morphologies of films fabricated via dry heterostructured electrified jets.

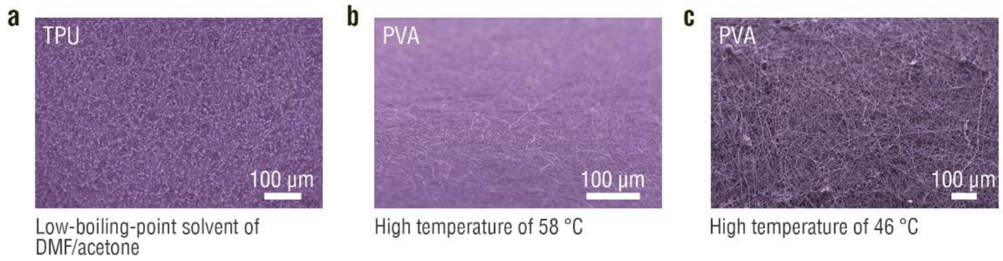

Supplementary Figure 13 Morphologies of films fabricated via dry heterostructured electrified jets. **a** SEM image of the TPU film fabricated by using low-boiling-point solvent. **b** SEM image of the PVA film fabricated by using water as the solvent at a high temperature of 58 °C. **c** SEM image of the PVA film fabricated by using a solvent consisting of water and acetone at a temperature of 46 °C.

2.14. Gas permeability and biocompatibility measurements.

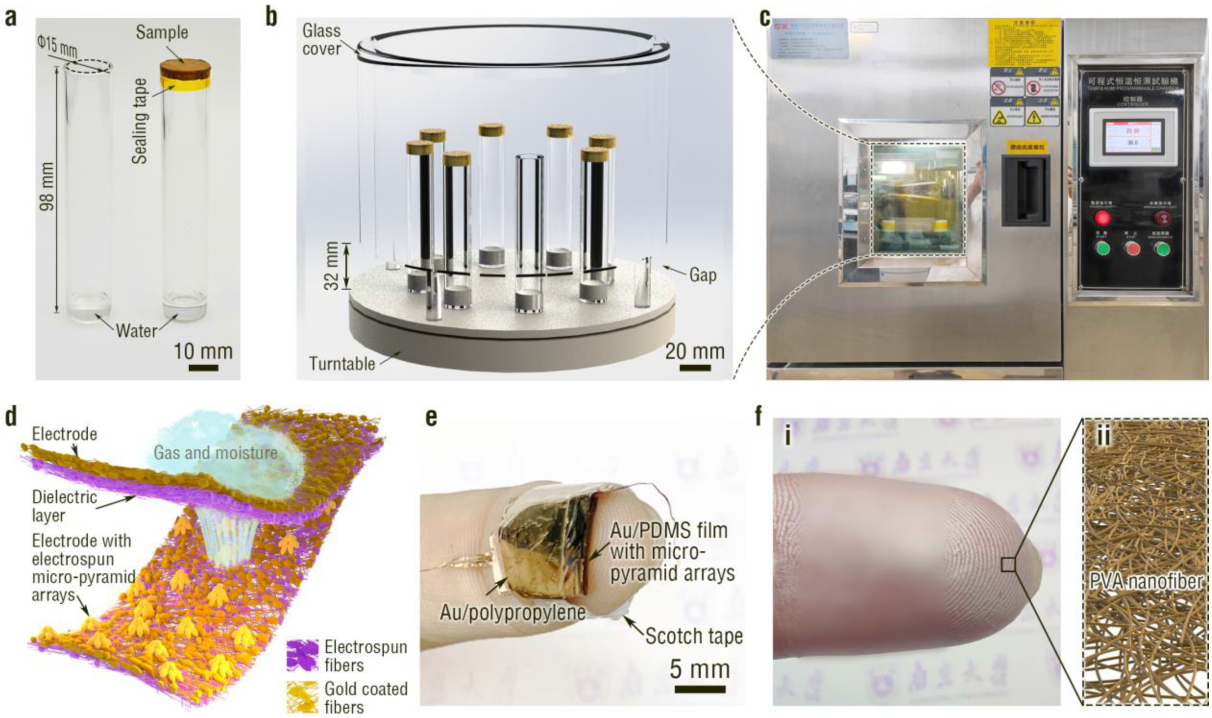

Supplementary Figure 14 Gas permeability and biocompatibility measurements. a Photograph of an open glass bottle and a glass bottle covered by a sample in the gas permeability test. **b, c** Picture of a constant climate chamber (c) and its internal set-up (b) in the gas permeability test. **d** Schematic diagram of the EMPA-based on-skin device. **e** Photographs showing a conventional PDMS micropyramid-array-based bilayer device attached to the fingertip for 7 h. **f** Electrospun PVA nanofibers deposited on the finger. (i) Photograph of the finger wrapped with electrospun PVA nanofibers. (ii) Schematic illustration of electrospun PVA nanofibers. Electrospun PVA nanofibers were deposited on the finger to attach the EMPA-based piezocapacitive sensor to the fingertip.

2.15. Slip ratio experiment.

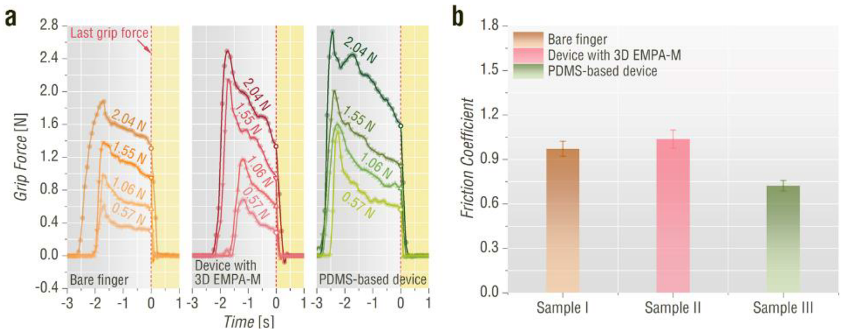

Supplementary Figure 15 Slip ratio experiment. a Last grip force of a participant with and without different devices attached to the finger. The yellow-shaded area means that no grip force acts on the object. **b** Calculated friction coefficient between the finger and the object under different surface conditions. Error bars are the standard error of the mean.

2.16. Optical properties, test setup and radiative cooling performance of electrospun films.

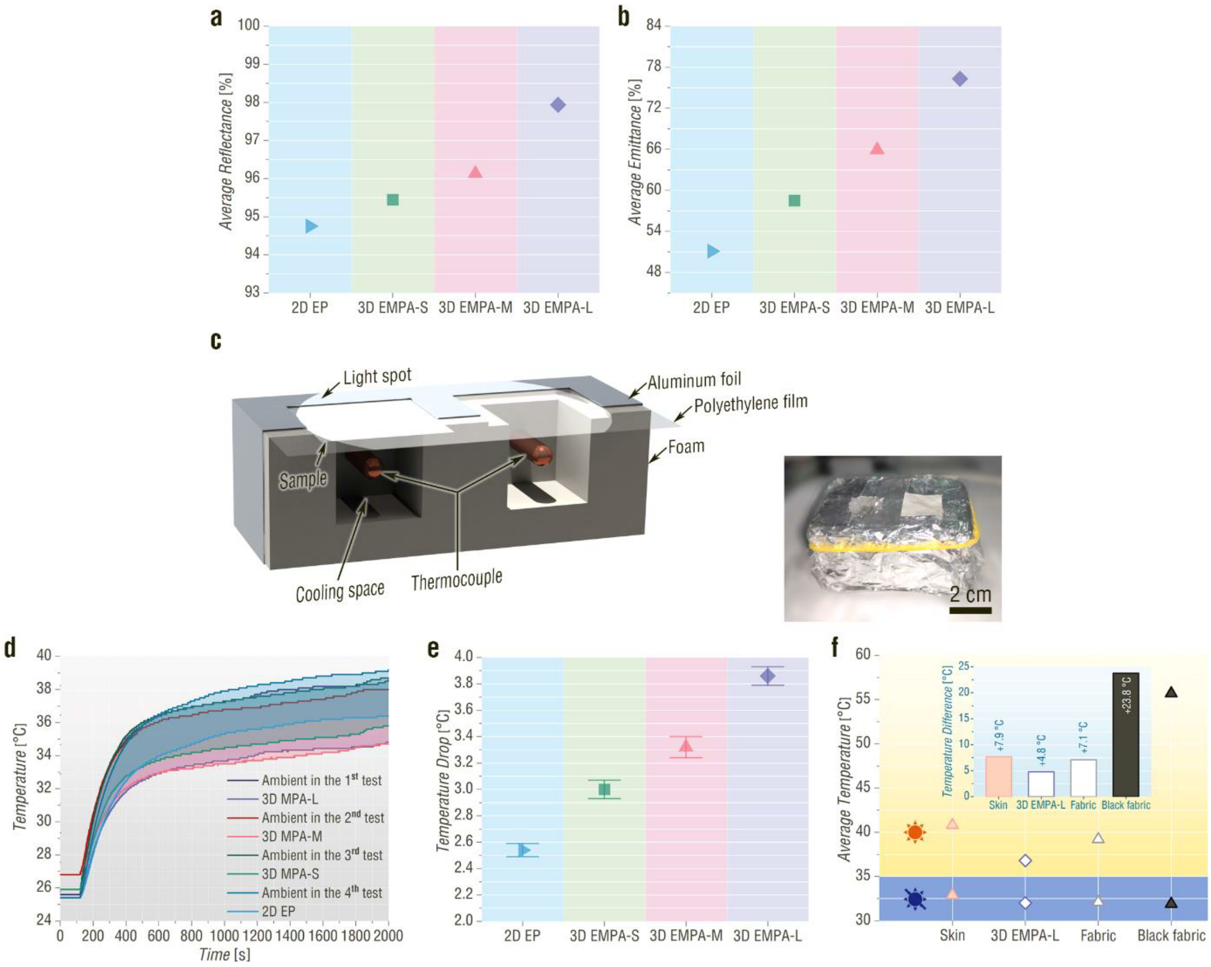

Supplementary Figure 16 Optical properties, test setup and radiative cooling performance of electrospun films. a Vis-NIR reflectivities of 2D EP, 3D EMPA-S, 3D EMPA-M, and 3D EMPA-L. **b** MIR emissivities of 2D EP, 3D EMPA-S, 3D EMPA-M, and 3D EMPA-L. **c** Schematic diagram (left) and photograph (right) of the cooling performance measurement. Two same foam cavities are under solar illumination with the same intensity of 1 kW m^{-2}. The one was covered with the sample. By contrast, the other exposed to solar illumination was the reference. The foam can decrease thermal conduction. The polyethylene film with high IR transparency and aluminum foil were used to avoid convection and reflect sunlight, respectively. **d** Temporal temperature profiles measured for 3D EMPA-L, 3D EMPA-M, 3D EMPA-S, and 2D EP. **e** Average temperature drops calculated according to

five identical daytime radiative cooling fabrics for each type of electrospun film. **f** Average temperatures of the skin, 3D EMPA-L, white cotton-containing fabric, and black cotton-containing fabric pasted up on the opisthenar before and after solar irradiation for about 8 min. The inset shows the average temperature rise of the skin, 3D EMPA-L, white cotton-containing fabric, and black cotton-containing fabric pasted up on the opisthenar before and after solar irradiation for about 8 min. The aforementioned white fabric contains 55% cotton cellulose and 45% polyester. The white fabric was dyed black by ink to obtain the black cotton-containing fabric.

2.17. Electrical characterization of EMPA-based piezocapacitive sensors.

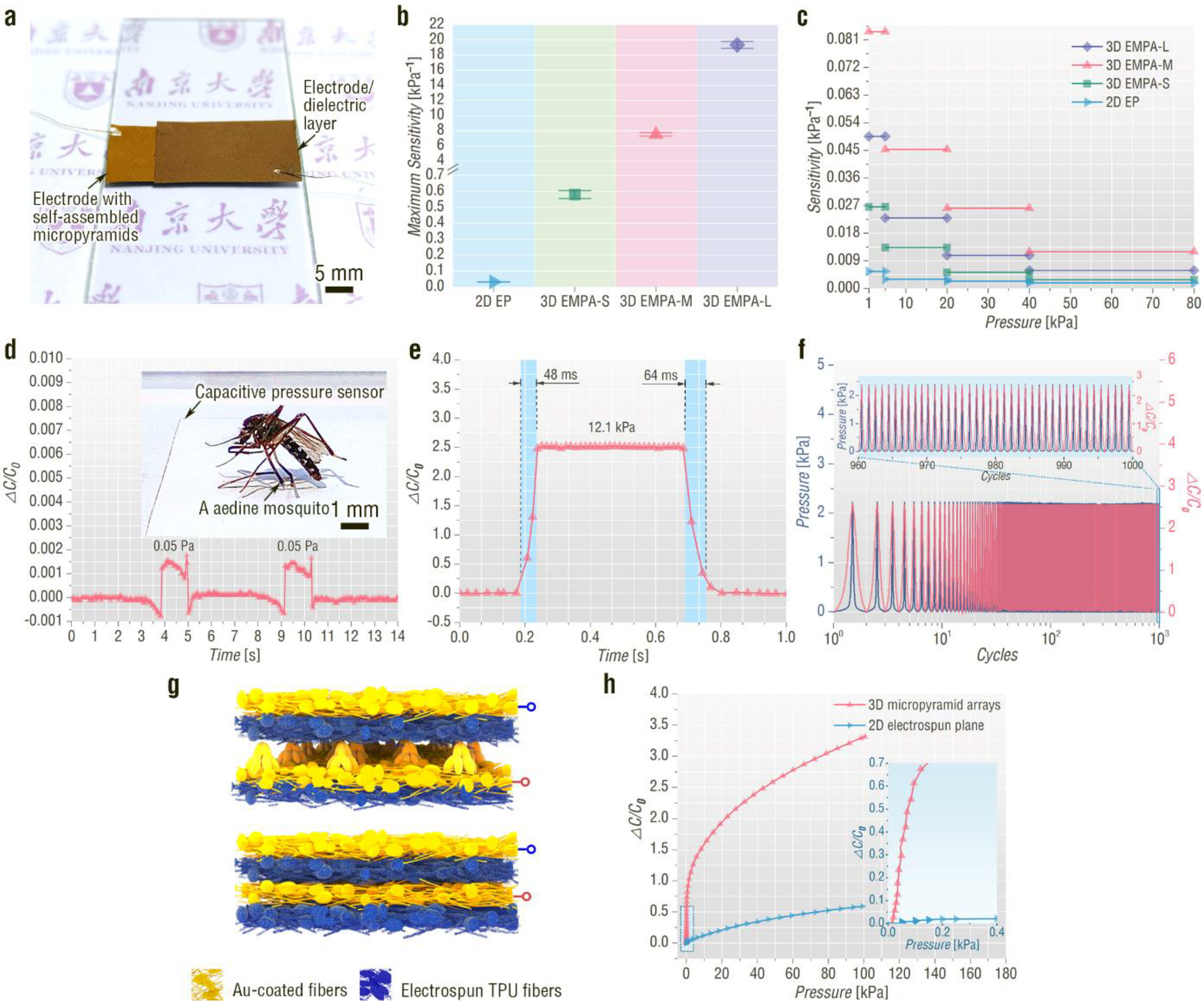

Supplementary Figure 17 Electrical characterization of EMPA-based piezocapacitive sensors. a Photograph of an EMPA-based piezocapacitive sensor. **b** Average maximum sensitivities calculated according to five identical piezocapacitive sensors for each type of electrospun film. **c** Sensitivities of piezocapacitive sensors based on the electrospun films with different average pyramid heights at the pressure above 1 kPa. **d** Detection limit of the 3D EMPA-M-based piezocapacitive sensor. The inset shows a 0.5 mg aedine mosquito landing on the EMPA-based piezocapacitive sensor. **e** Response time of the 3D EMPA-M-based piezocapacitive sensor when pressing and releasing. The pressure value in the response time test is 12.1 kPa. **f** Dynamic capacitance change of the 3D EMPA-M-based piezocapacitive sensor during 1000 cyclic pressure applications. The inset represents the cycle

ranging from 961 to 1000. **g, h** Sensing performance of TPU-based piezocapacitive sensors. **g** Schematic diagram showing the structures of piezocapacitive sensors based on TPU EMPA and flat TPU electrospun film, respectively. **h** Relative capacitance change as a function of pressure applied to TPU-based piezocapacitive sensors. The inset presents the pressure range from 0 to 0.4 kPa.

2.18. Working mechanism, test setup and electrical performance of electrospun film-based TENGs.

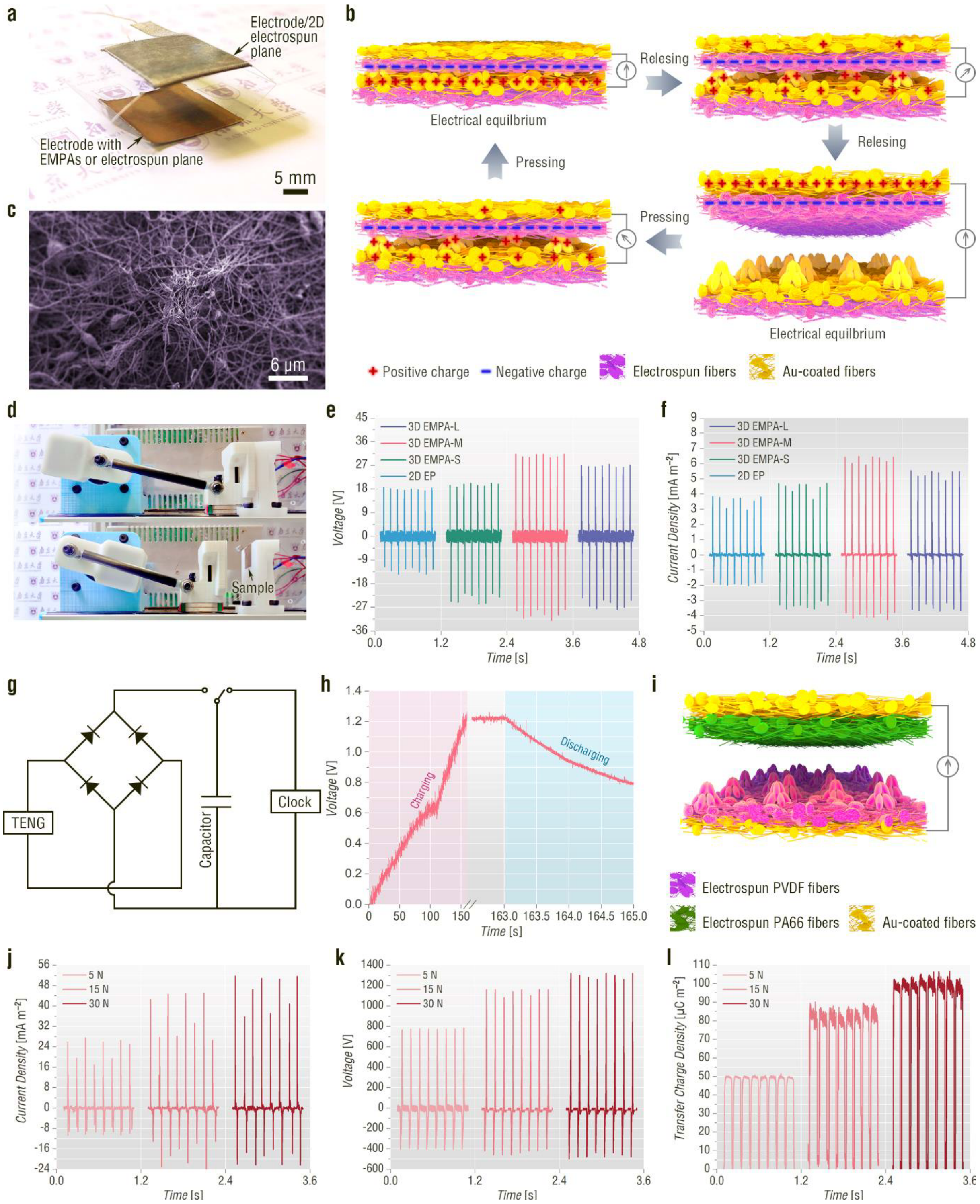

Supplementary Figure 18 Working mechanism, test setup and electrical performance of electrospun film-based TENGs. a Photograph of the TENG for output performance measurement. **b** Schematic illustration of the working mechanism for the EMPA-based TENG.

Our proposed TENGs feature an air gap between a tribopositive layer and a tribonegative layer made of Au-coated electrospun films and output alternating signals during a cyclic contact-separation motion of a pair of tribolayers owing to contact electrification and electrostatic induction[4-10]. **c** SEM image of a depolarized electrospun micropyramid. The PVDF electrospun films were depolarized by annealing at 150 °C for 24 h to exclude piezoelectric interference from in-situ polarization of electrospinning and concurrently keep micromorphology unchanged. **d** Depiction of the custom-made TENG test apparatus with a step motor linked to a slider by means of a connecting rod. **e, f** Electrical outputs of depolarized electrospun film-based TENGs with an Au-coated 2D EP as the tribopositive layer. (**e**) Output voltages and (**f**) short-circuit current densities of the TENGs based on 3D EMPA-L, 3D EMPA-M, 3D EMPA-S, and 2D EP. All samples were depolarized by temperature aging to avoid interference from the piezoelectric effect. The impact force acting on the device is 5 N. **g** Circuit diagram of charging a 10 μF capacitor and driving a digital clock by the 3D EMPA-M-based TENG. **h** Charging and discharging curve in the biomechanical energy harvesting experiment. **i** Schematic diagram of the EMPA TENG consisting of a 30-μm-thick flat Au-coated PA66 electrospun film and a 3D EMPA-M (i.e., the improved TENG). This type of TENG serves as a high-efficiency mechanical energy harvester. **j–l** Electrical outputs of the improved TENG. (**j**) Current density, (**k**) output voltage, and (**l**) transfer charge density of the improved TENG under different impact forces.

2.19. Energy conversion efficiency test of TENGs.

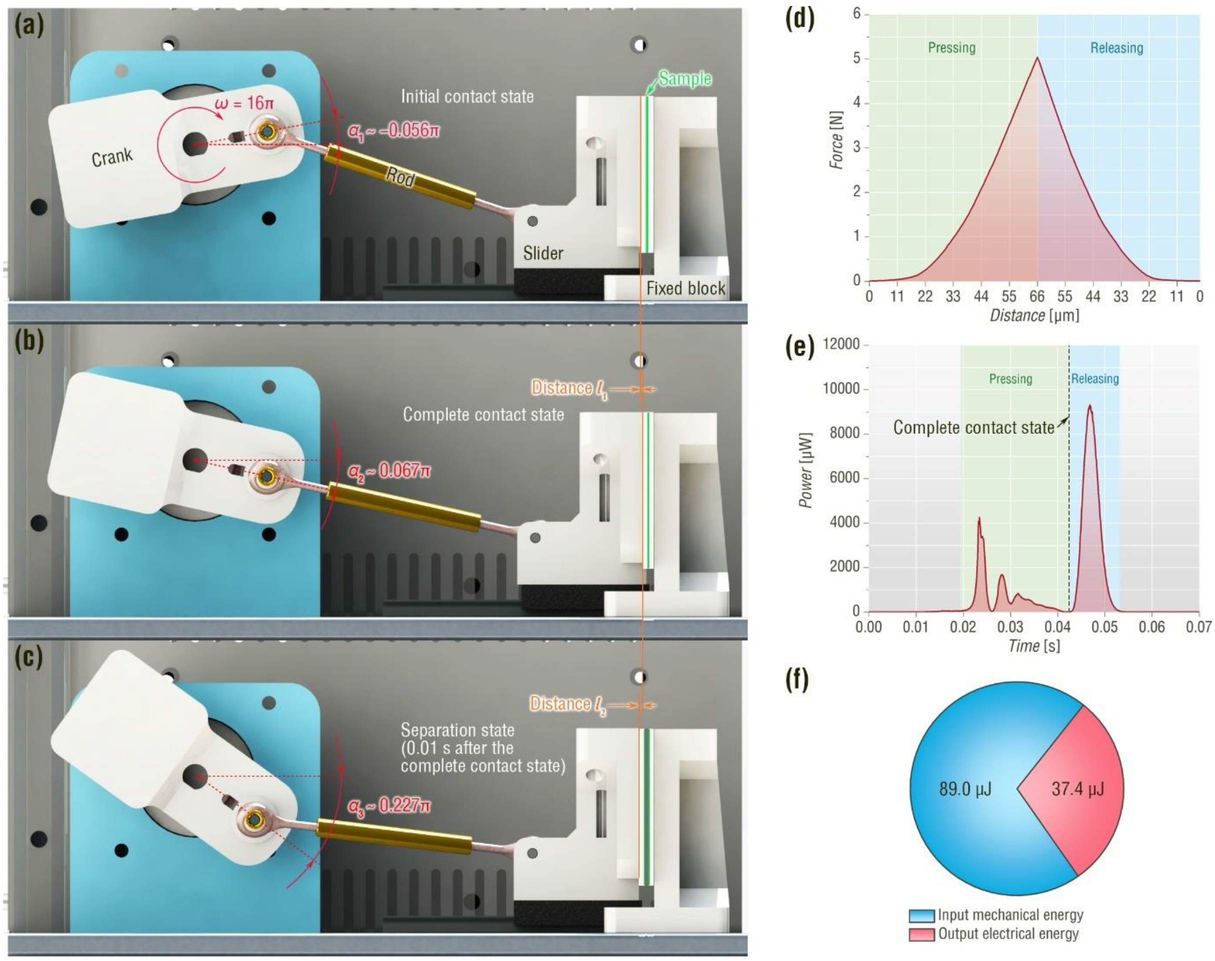

Supplementary Figure 19 Energy conversion efficiency test of TENGs. a Schematic illustration of the initial contact state. **b** Schematic illustration of the complete contact state. **c** Schematic illustration of the separation state (i.e., 0.01 s after the complete contact state). **d** Applied force towards the improved TENG as a function of distance. **e** Output power of the improved TENG with the best matched external load of 50 MΩ during the pressing and releasing processes. **f** Average values of the input mechanical energy and the output electrical energy of the improved TENG after replications of five sets of experiments.

2.20. Estimation of the biomechanical-to-electrical energy conversion efficiency.

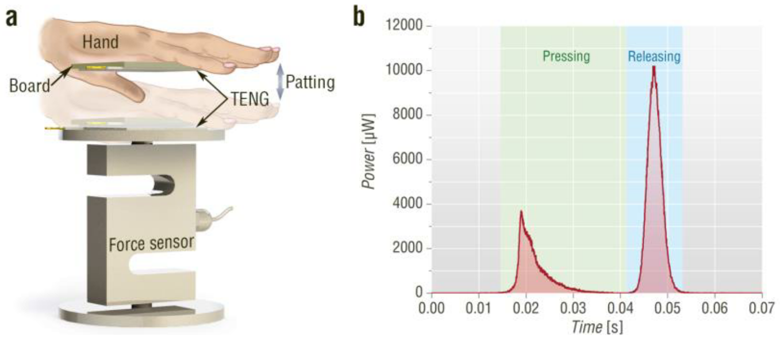

Supplementary Figure 20 Estimation of the biomechanical-to-electrical energy conversion efficiency. a Schematic diagram of the set-up for estimating the bioenergy harvesting efficiency. **b** Output power in the bioenergy harvesting efficiency measurement.

2.21. Characterization of EMPA-based piezoelectrics and corresponding PENGs.

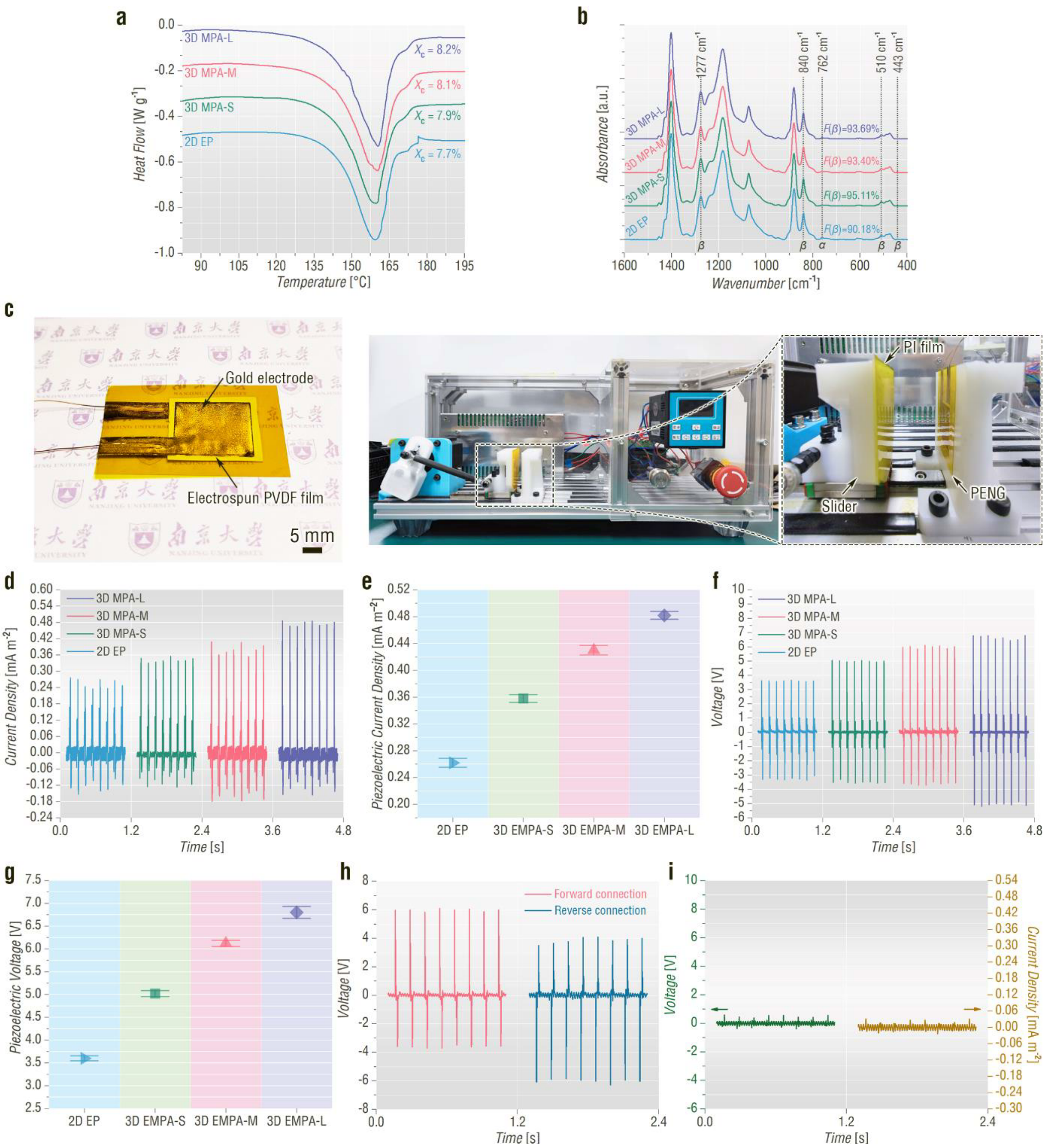

Supplementary Figure 21 Characterization of EMPA-based piezoelectrics and corresponding PENGs. a DSC traces and percentage crystallinities (X_c) of pristine 3D EMPA-L, 3D EMPA-M, 3D EMPA-S, and 2D EP. The X_c of electrospun PVDF films were calculated according to the equation, $X_c = (\Delta H_m / \Delta H_m^0) \times 100\%$, where ΔH_m is the melting enthalpy of various electrospun PVDF films, and ΔH_m^0 is the melting enthalpy value of the 100% crystalline PVDF (i.e., 104.7 mJ mg^{-1})[11]. The ΔH_m values of 3D EMPA-L, 3D EMPA-

M, 3D EMPA-S, and 2D EP from the DSC heating curves are 8.57, 8.51, 8.24, and 8.09 mJ mg^{-1}, respectively. **b** FT-IR spectra and β-phase contents ($F(\beta)$) of pristine 3D EMPA-L, 3D EMPA-M, 3D EMPA-S, and 2D EP. The $F(\beta)$ of electrospun PVDF films were calculated according to the equation, $F(\beta) = A_\beta / (1.26A_\alpha + A_\beta)$, where A_α and A_β are the absorbance intensities at 762 and 840 cm^{-1} [12]. **c** Photographs of the PENG and the apparatus for output performance measurement. **d–g** Electrical outputs of pristine electrospun-film-based PENGs under an impact force of 5 N. (**d**) Current densities, (**e**) average current densities for five identical samples, (**f**) output voltages, and (**g**) average current densities for five identical samples of the PENGs based on pristine 2D EP, 3D EMPA-S, 3D EMPA-M, and 3D EMPA-L. **h, i** Verification of the actual piezoelectric signals. (**h**) Measured voltages in forward and reverse connection modes. (**i**) Voltage and short-circuit current density of the TENG based on a depolarized 3D EMPA-M. The authenticity of the piezoelectric signals was verified by both the same magnitude signals under the forward and the reverse connection modes and the faint output of the depolarized sample.

2.22**. Fingertip pulse waveforms detected by different devices.**

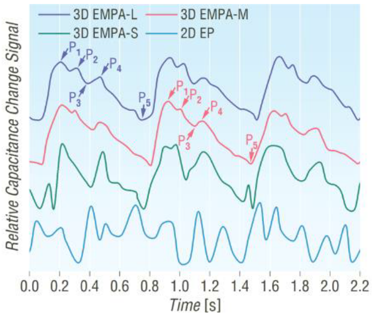

Supplementary Figure 22 Fingertip pulse waveforms detected by different devices. Fingertip pulse waveforms detected by 3D EMPA-L, 3D EMPA-M, 3D EMPA-S, and 2D EP piezocapacitive sensors, respectively.

2.23. Working mechanism of EMPA-based triboelectric sensor attached to a mouse.

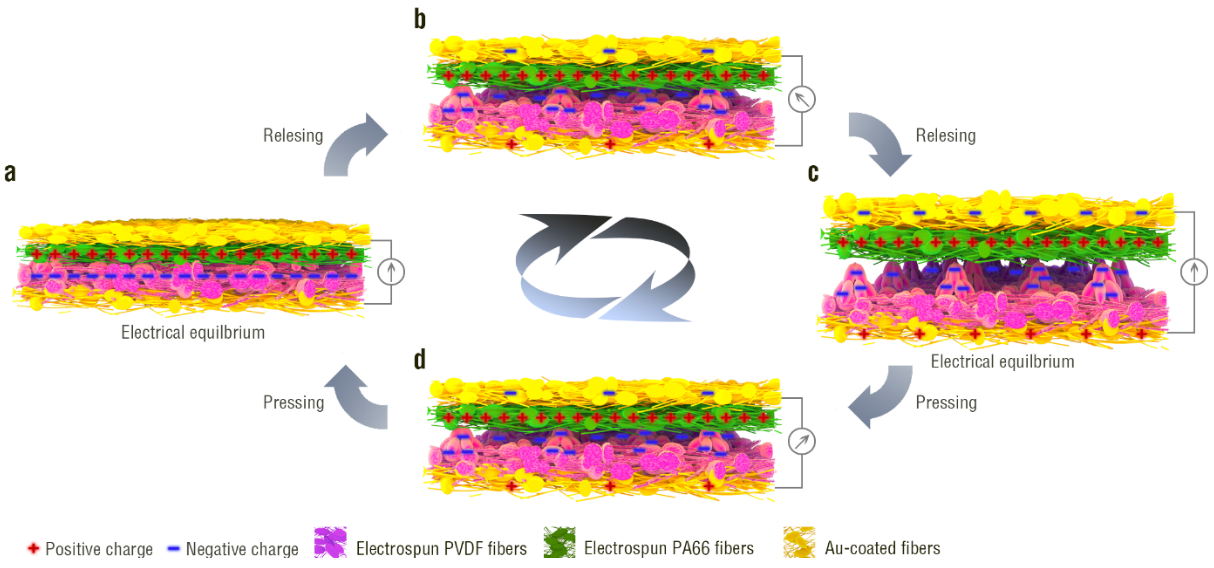

Supplementary Figure 23 Working mechanism of EMPA-based triboelectric sensor attached to a mouse. Schematic illustration of the working mechanism for the EMPA-based TENG Sensor II.

2.24. Electrical characterizations of triboelectric and piezocapacitive sensors.

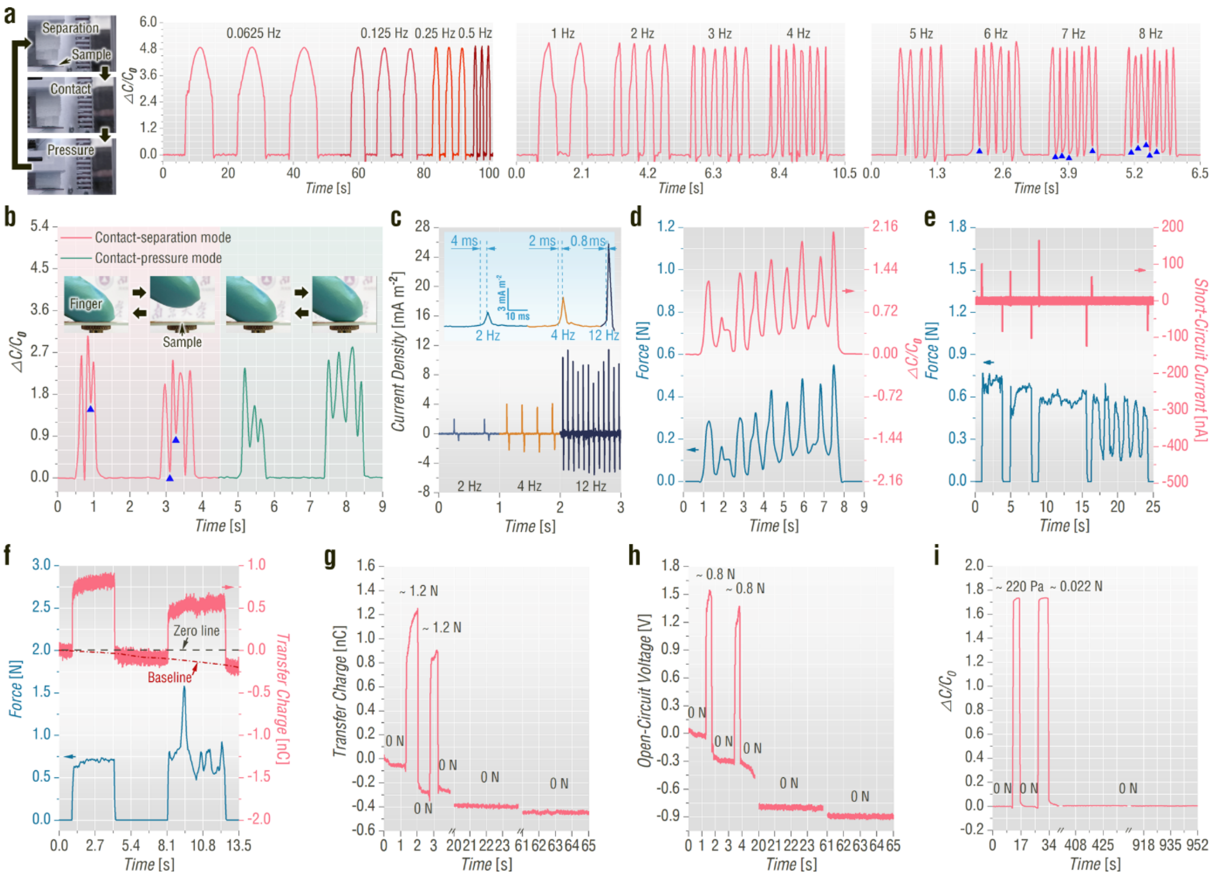

Supplementary Figure 24 Electrical characterizations of triboelectric and piezocapacitive sensors. a Photographs of cyclic separation, contact, and pressed states during the test process and relative capacitance change detected at a fixed maximum instantaneous pressure of about 200 kPa and at the frequency ranging from 0.0625 to 8 Hz. **b** Relative capacitance change in the contact-separation and contact-pressure modes. The insets show the contact-separation mode (left) and the contact-pressure mode (right). The blue triangles stand for the misidentification points due to the long response time of the piezocapacitive sensor. **c** Short-circuit current density of the triboelectric sensor detected at the frequency ranging from 2 to 12 Hz. The inset shows the ultrafast response of the triboelectric sensor. **d** Dependence of relative capacitance change of the piezocapacitive sensor on applied force. **e** Dependence of transfer charge of the triboelectric sensor on applied force. **g, h** (**g**) Transfer charge and (**h**) open-circuit voltage of the triboelectric sensor

undergoing gentle finger pressing twice. (**i**) Relative capacitance change of the piezocapacitive sensor undergoing gentle finger pressing twice.

2.25. PRV test.

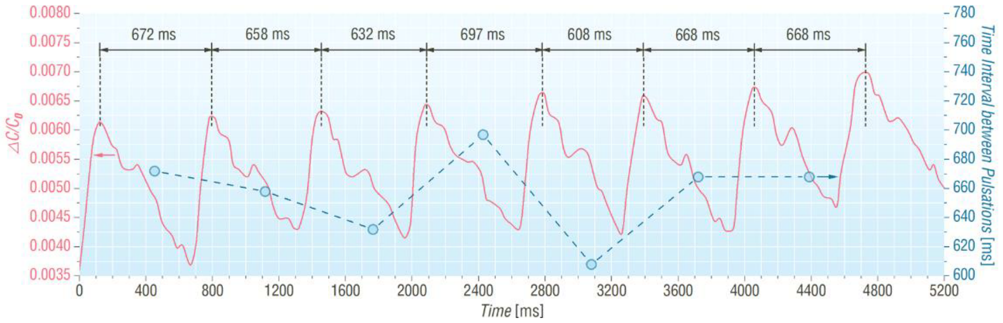

Supplementary Figure 25 PRV test. Diagrammatic sketch of PRV measured via the 3D EMPA-M-based on-skin piezocapacitive sensor attached to a fingertip. PRV is the physiological phenomenon of variation in the time interval between pulses. It is tested by the variation in the beat-to-beat interval. It can be used to analyze emotion, mental state, physical condition, and so on.

3. Supplementary Notes.

Supplementary Note 1: Formation process of wet heterostructured jets.

In this work, two kinds of wet heterostructured jets (i.e., the bead-on-string PVDF, TPU or PVA jets and the fibrous PVA jets with uneven diameter (Supplementary Fig. 3c and 12)) can spontaneously compose EMPAs. Their formation process is as follows. The joint effect of electrostatic force and surface tension drives the jet to eject from the Taylor cone forming at the spinneret tip[13]. When surface tension exerts a greater influence on jets than Coulombic repulsive force, electrified jets with nonuniform specific surface areas (i.e., heterostructured jets) are apt to form[7,14,15]. Herein, a series of selective dilute solutions with low viscosity are used to enhance the surface tension acting on jets[5] and to obtain heterostructured jets with appropriate size as well as composition. Meanwhile, various solvents (DMSO and water) with low saturated vapor pressure (i.e., high boiling point) are used to ensure incomplete volatilization of the solvent just as the jets come into contact with the collector. Therefore, the jet region with low specific surface area, such as the bead part, is wet.

Supplementary Note 2: Formation process of differently charged initial fibers.

The electrospinning of wet heterostructured electrified jets can lead to differently charged initial fibers deposited on a collector. The deposited microdomains containing less PVDF carry positive charges, but the deposited microdomains containing more PVDF are negatively charged[7,14]. The formation process of these differently charged initial fibers is shown in Supplementary Fig. 4. When the first batch of positively charged bead-on-string PVDF jets eject from the spinneret tip and crash against the negatively charged aluminum foil electrically grounded, positive charges will be remaining on the string parts for a time due to their high electrical insulation[16,17]. In contrast, it is hard for the initially deposited bead parts with low insulation to continue binding positive charges. Under the effect of the electric field in the electrospinning machine, the initially deposited bead parts are easy to be polarized[18,19], and therefore negative charges appear on the top surface of bead parts (Supplementary Fig. 4c). Subsequently, the deposited microdomains enriched with positive charges repel the next batch of positively charged jets[20]. On the contrary, the aerial jets are attracted by the deposited microdomains with negative charges (Supplementary Fig. 4c),[19] composing new thicker negatively charged microdomains.

Additionally, the charged situation of the collector and the insulation difference between the string parts and the bead parts are further illustrated by the following two sections. Firstly, to determine the charged situation of the collector, we tested the surface potential of the aluminum foil (i.e., the collector). As shown in Supplementary Fig. 5a, when the voltage of the power supply in the electrospinning machine is zero, the surface potential of the aluminum foil is zero. When the voltage reaches up to +12.5 kV, the surface potential of the aluminum foil is –4.3 kV (Supplementary Fig. 5b). It is concluded that net negative charges appear on the surface of the aluminum foil under the effect of the electric field at the beginning of electrospinning.

Secondly, the insulation difference was experimentally demonstrated. For ease of description, the string part and the bead part of the bead-on-string fiber are designated as “nanowire” and “microsphere”, respectively. In the process of electrospinning, the electrified jets are ejected from the spinneret and then accelerated by the electric field. The aerial jets subjected to the electric field force are elongated and thinned[21]. During this process, the solvent in the string-shaped regions of the jets completely evaporates or evaporates in a large amount due to their high specific surface area, resulting in a huge percentage of solute in the deposited nanowires. Moreover, the solute (i.e., PVDF) used in this work has excellent electrical insulation. Therefore, the initially deposited nanowires possess high insulation. In contrast, more solvent remains in the deposited microspheres because their low specific surface area and low saturated vapor pressure of the solvent[6] limit solvent evaporation. Consequently, initially deposited microspheres have low insulation. Note that DMSO with a high boiling point of 189 °C[22] is used as a component of the solvent.

A weighing experiment can indicate that partial solvent still remains in initially deposited microspheres. Compared with the fresh EMPA films at the end of electrospinning, the EMPA films dried at room temperature for more than 6 h exhibited significant decrease in weight. For example, the mass of the 3D EMPA-M films is reduced by approximately 70% after drying.

To verify the insulation difference between the initially deposited nanowires and microspheres, we tested the resistance of an electrospun PVDF film before and after wetting with the solvent DMSO. The experimental procedure is shown in Supplementary Fig. 6. An ultrathin dry PVDF film (thickness ~ 5 μm) was deposited on a smooth flat titanium (Ti) plate by electrospinning (Supplementary Fig. 6a, b). Subsequently, another smooth flat Ti plate was placed on top of the electrospun-film-deposited Ti plate. With two Ti plates as electrodes, the resistance of the dry electrospun film was tested (Supplementary Fig. 6c). When the centre of the electrospun film was wetted with DMSO, the film resistance was tested as well. The

resistances of the electrospun film before and after wetting treatment are 759077 and 0.1397 kΩ, respectively, which reflects the remarkable insulation difference.

Supplementary Note 3: Necessity of the wet heterostructured electrified jets for fabricating EMPAs.

By virtue of wet rather than dry heterostructured electrified jets, a variety of EMPAs are successfully fabricated (Fig. 1c and 2c). When we choose low-boiling-point solvents or raised temperature during electrospinning to make the deposited heterostructured jets dry, only flat electrospun films are prepared (Supplementary Fig. 13). Note that other experimental conditions remained unchanged. This result indicates the necessity of the wet heterostructured electrified jets.

Supplementary Note 4: The reason why negative charges appear on the tops of electrospun microdomes and micropyramids, and the reason why the local electric field is higher at a position closer to the tip of the microdome or micropyramid.

Electrostatic induction and polarization under the effect of the electric field in the electrospinning machine lead to the appearance of negative charges on the tops of electrospun micro-protuberances (i.e., microdomes and micropyramids)[19]. As shown in Supplementary Fig. 7, the surface potential value of the deposited EMPA film during electrospinning is a minus, which indicates that the film surface facing the spinneret is macroscopically electronegative. Obviously, there must be microdomains that are negatively charged. SEM images and statistical data in Supplementary Fig. 8 show that the position closer to the top of a micro-protuberance has a higher fiber density, and the fibers densely accumulate at the tip of a micro-protuberance. Since positively charged aerial jets tend to deposit on the microdomains carrying more negative charges, it is concluded that there are negative charges at the tops of microdomes and micropyramids.

Finite element simulation was used to illustrate the local electric fields at different positions for the electrospun micro-protuberances. As shown in Supplementary Fig. 9, whether for an electrospun microdome or an electrospun micropyramid, the position closer to the tip of the micro-protuberance has a higher local electric field.

Supplementary Note 5: Dependence study between voltage and size of EMPAs.

We control the voltage to adjust the size, composition, and charge density of heterostructured jets. As the voltage increases from 10.0 to 17.5 kV, the micropyramid arrays first appear, then dwindle in size, and finally disappear. When the voltage is only 10.0 kV, both the quantity ratio of beads to springs and the charge density on jet surfaces are too low, failing to meet the self-assembly conditions (Supplementary Fig. 10a-i). When the voltage is 12.5 kV, the fiber size and the quantity ratio of beads to springs reach the best. Typical EMPAs are successfully fabricated (Supplementary Fig. 10a-ii). A higher voltage of 15.0 kV makes parameters involving the jets deviate from optimum values, causing the smaller micropyramid size (Supplementary Fig. 10a-iii). Once the voltage reaches up to 17.5 kV, oversized bead-like jets make the self-assembly impossible (Supplementary Fig. 10a-iv).

Supplementary Note 6: Removal of the interaction between the finger and the object.

In the object-grasping experiment, surface material ($P < 0.001$), load force ($P < 0.001$) and interaction between the finger and the object ($P < 0.001$) are the main determinants of the grip force. To remove the effect of interaction on the grip force, we subtract the minimum grip force necessary (i.e., the last grip force, Supplementary Fig. 15a) based on the grasp friction coefficient (Supplementary Fig. 15b) and express the amount of grip force exceeding this level (i.e., additional grip force, Fig. 3h).

Supplementary Note 7: Purpose of the experiment about the EMPA films with different APHs.

The precise structure–performance relationships of the micropyramid-arrayed devices in terms of daytime radiative cooling[23], piezocapacitive sensing[24-26], and energy harvesting based on triboelectric[27-29] and piezoelectric[30] effects have been systematically studied and demonstrated in previous classic works. The performance comparison among the devices with different APHs in this work is a proof of concept according to the findings of previous works. The purpose of the experiment about the EMPA films with different APHs is to highlight the importance of the structural tunability. The corresponding device performance can be further optimized thanks to the structural tunability of the flexible self-assembly technology. The main contribution of this work is exploring the formation factors, growth process, designability, and promising applications of such an innovative EMPA structure rather than carrying out a precise structure–performance relationship study.

Supplementary Note 8: Energy conversion efficiency of the improved TENG.

The energy conversion efficiency (η) of the nanogenerator is defined as the ratio of the output electrical energy transferred to the external load and the input mechanical energy[31]. The input mechanical energy consists of (i) the work done by a compression force between the initial contact state and the complete contact state as well as (ii) the work done by a force separating a pair of tribolayers.

A custom-made apparatus based on offset slider crank mechanism was first used to determine the mechanical-to-electrical energy conversion efficiency. When the angle between the horizon and the crank (α) is approximately −0.056π, a pair of tribolayers start touching each other (i.e., initial contact state, Supplementary Fig. 19a). When the α is approximately 0.067π, tribolayers are in full contact (i.e., complete contact state, Supplementary Fig. 19b). The work done between the initial contact state and the complete contact state (W_i) can be expressed as:

$$W_i = \int F_c \, dl \tag{2}$$

where F_c is the compression force, l is the moving distance of the slider. l_1 in Supplementary Fig. 19b is the moving distance of the plane between the initial contact state and the complete contact state. Supplementary Fig. 19d shows the F_c–l curve when a TENG consisting of a 3D EMPA-M and a flat electrospun PA66 film (i.e., the improved TENG) is sandwiched between the slider and the fixed block. The calculated W_i is approximately 89 μJ. Supplementary Fig. 19e shows the output power curve of the improved TENG. The electrical energy of the TENG is completely released at 0.01 s after the complete contact state (i.e., separation state, Supplementary Fig. 19c). At this time the α is approximately 0.227π, and the distance between the separation state and the complete contact state (l_2) is approximately 140 μm. The work done by a force separating a pair of tribolayers (W_{ii}) can be expressed as:

$$W_{ii} = \int F_s \, dl \tag{3}$$

where F_s is the force that enables a pair of tribolayers to separate from each other.

$$F_s = m\,a \tag{4}$$

where m is the mass of the tribolayer attached to the slider, and a is the acceleration of the tribolayer attached to the slider.

$$a = -\omega^2 r\left(\cos\alpha + \frac{r}{L}\cos 2\alpha - \frac{e}{L}\sin\alpha\right) \tag{5}$$

where ω is the angular velocity of the crank, r is the radius of the crank, L is the length of the rod, and e is the offset. l_2 is put into Supplementary Eq. 3 to evaluate the required W_{ii} as high as possible. The calculated W_{ii} is less than 0.15 μJ, which is so small compared to W_i that it can be negligible. Therefore, η can be evaluated according to the following equation:

$$\eta\% = \frac{\int \frac{U^2}{R}\mathrm{d}t}{W_i} \tag{6}$$

where U is the output voltage, R is the resistance of the best matched external load (1–50 MΩ), and t is the time.

Supplementary Fig. 19f shows the average values of the input mechanical energy and the output electrical energy of the improved TENG after replications of five sets of experiments. Its energy conversion efficiency reaches up to 42% ± 1%.

The biomechanical-to-electrical energy conversion efficiency of the improved TENG is also estimated by using the same approach. Supplementary Fig. 20a shows the set-up of the measurement. The tribopositive and tribonegative layers are respectively attached to a force sensor and a board that is fixed on a hand. The force and electrical signals are recorded when the subject pats the force sensor (Supplementary Fig. 20b). The calculated bioenergy harvesting efficiency is 41%.

Supplementary Note 9: Drawbacks of the commercial photoplethysmograph.

In terms of the commercial photoplethysmograph, a series of drawbacks stem from its hard as well as heavy structure and working mechanism of photoplethysmography. If the photoplethysmograph is placed so tightly on the person, ischemic pressure necrosis may occur. Prolonged placement of a photoplethysmograph may result in mechanical injuries, such as finger stiffness, making it difficult for the person to flex the finger after the photoplethysmograph has been removed. In addition, nail polish which absorbs light from diodes may interfere with the photoplethysmograph. The aforementioned complications have been documented[32].

4. Supplementary Tables.

Supplementary Table 1 Materials for fabricating various electrospun films.

Name	CAS No.	Manufacturer	Purity	Product No.	Remark
Poly(vinylidene fluoride) (PVDF)	24937-79-9	Sigma-Aldrich®	No datum	182702	Average M_w ~534000, powder
Thermoplastic polyurethane (TPU)	No datum	Bayer®	No datum	9370AU	Pellets
Poly(vinyl alcohol) 1788 (PVA)	9002-89-5	Aladdin®	No datum	P105124	Powder
Poly(hexamethylene adipamide) (PA66)	32131-17-2	Sigma-Aldrich®	No datum	429171	Pellets
Dimethyl sulfoxide (DMSO)	67-68-5	Aladdin®	≥99.9% (GC)	D103277	Boiling point ~189 °C
Acetone	67-64-1	Hushi®	≥99.5% (AR)	10000418	Boiling point ~57 °C
N,N-dimethylformamide (DMF)	68-12-2	Aladdin®	>99.9% (GC)	D112002	Boiling point ~153 °C
Deionized water	7732-18-5	Macklin®	≥99.9975%	W820537	Boiling point =100 °C
Hexafluoroisopropanol (HFIP)	920-66-1	Aladdin®	99.5% (CP)	H107501	Boiling point ~59 °C
Formate	64-18-6	Aladdin®	≥98% (GC)	F112034	Boiling point ~101 °C
Smooth aluminum foil	7429-90-5	Titan®	02038479	TYJS-LB	Thickness ~15 μm

It is noted that all materials were used without any further purification.

Supplementary Table 2 Electrospinning parameters for fabricating various electrospun films.

Sample	Solvent	Solute	Positive high voltage [kV]	Distance between needle and collector [mm]	Solution flow rate [mL h^{-1}]	horizontal swing distance [mm]	Solution volume [mL]	Tempera-ture [°C]	Relative humidity [%]
3D EMPA-L (Fig. 1c, 2a-II, 2b-i, Supplementary Fig. 10a-ii, 10b-i, 11a-i, and 11b-i)	DMSO and Acetone (volume ratio of 2∶1)	PVDF (8.59 wt%)	12.5	80	1.0	40	5.42	25±2	40±3
3D EMPA-M (Fig. 2b-ii, Supplementary Fig. 10b-ii, 11a-ii, 11b-ii, and 18c)	DMSO and Acetone (volume ratio of 2∶1)	PVDF (8.59 wt%)	12.5	80	1.0	40	5.42	25±2	60±3
3D EMPA-S (Fig. 2b-iii, Supplementary Fig. 10a-iii, 10c-i, 11a-iii, and 11b-iii)	DMSO and Acetone (volume ratio of 2∶1)	PVDF (8.59 wt%)	15.0	80	1.0	40	5.42	25±2	40±3
2D EP (Fig. 2b-iv, Supplementary Fig. 10a-i, 11a-iv, and 11b-iv)	DMSO and Acetone (volume ratio of 2∶1)	PVDF (8.59 wt%)	10.0	80	1.0	40	5.42	25±2	40±3
Gradually thickened electrospun film with growing size of pyramid arrays (Fig. 2a, Supplementary Fig. 2d and 2e)	DMSO and Acetone (volume ratio of 2∶1)	PVDF (8.59 wt%)	12.5	80	1.0	40	5.42	25±2	40±3
Electrospun PVDF film fabricated under the humidity of 80% (Supplementary Fig. 10b)	DMSO and Acetone (volume ratio of 2∶1)	PVDF (8.59 wt%)	12.5	80	1.0	40	5.42	25±2	80±3
Electrospun PVDF film fabricated under the voltage of 17.5 kV (Supplementary Fig. 10a-iv)	DMSO and Acetone (volume ratio of 2∶1)	PVDF (8.59 wt%)	17.5	80	1.0	40	5.42	25±2	40±3
Electrospun PVDF film fabricated with horizontal swing distance of 25 mm (Supplementary Fig. 10c-ii)	DMSO and Acetone (volume ratio of 2∶1)	PVDF (8.59 wt%)	15.0	80	1.0	0	5.42	25±2	40±3
Electrospun TPU micropyramid arrays (Fig. 2c-i and Supplementary Fig. 12a)	DMSO and Acetone (volume ratio of 1∶1)	TPU (20.9 wt%)	20.0	100	1.0	40	5.42	25±2	30±3
Flat electrospun TPU film (Supplementary Fig. 13a)	DMF and Acetone (volume ratio of 1∶1)	TPU (20.9 wt%)	20.0	100	1.0	40	5.42	25±2	30±3
Electrospun PVA micropyramid arrays (Fig. 2c-ii and Supplementary Fig. 12b)	Water and Acetone (volume ratio of 2∶1)	PVA (10.0 wt%)	15.0	61	0.5	40	3.00	25±2	60±3
Flat electrospun PVA film (Supplementary Fig. 13c)	Water and Acetone (volume ratio of 2 ∶ 1)	PVA (10.0 wt%)	15.0	61	0.5	40	3.00	46±2	30±3
Electrospun PVA micropyramid arrays (Supplementary Fig. 12c)	Water	PVA (10.0 wt%)	15.0	61	0.5	40	3.00	25±2	60±3
Flat electrospun PVA film (Supplementary Fig. 13b)	Water	PVA (10.0 wt%)	15.0	61	0.5	40	3.00	58±2	27±3
Flat electrospun PA66 film (Supplementary Fig. 13c)	HFIP	PA66 (4.23 wt%)	25.0	100	0.5	40	3.00	25±2	55±3
On-skin electrospun PVA nanofiber glue (Fig. 3c and Supplementary Fig. 14f)	Water	PVA (9.5 wt%)	15.0	50±20	0.5	0	0.025	40±2	30±3

It is noted that except above-mentioned parameters listed in Supplementary Tab. 2, other parameters for preparing various electrospun films are the same. The production speed of 3D-EMPA-L, 3D-EMPA-M, and 3D-EMPA-S is ~520 cm^2 h^{-1} by using a 5 mL spinneret.

Supplementary Table 3 VAS grading scale for biocompatibility study.

Score	Description
0	No sensory interference & no discomfort (Feeling perfectly normal)
1	Minor sensory interference or discomfort (Exerting no impact on work and daily life)
2	
3	
4	Moderate discomfort (Exerting little impact on daily life but exerting impact on work)
5	
6	
7	Severe discomfort (Exerting significant impact on work and daily life)
8	
9	
10	Extreme discomfort (Unable to engage in normal activities at work and in life)

Supplementary Table 4 Cooling performance comparison among the EMPA films and other gas-permeable fabrics reported in recent years.

Ref.	Sample	Structure	Thickness	Test conditions	Cooling temperature
Y. Cui's group, Science, 2016[33]	Polyethylene textile	Interconnected nanopores that are 50 to 1000 nm in diameter	12 µm	The heating element (power density 140 W m^{-2}) that generates constant heating power is used to simulate human skin.	2.7 °C lower than the skin covered with cotton.
Y. Cui's group, Nat. Sustain., 2018[34]	Polyethylene fabric	Nanoporous microfibers with a cavity size of 100–1000 nm	450 µm	A simulated skin with a heating power density of 95 W m^{-2} is covered with the textile sample.	2.3 °C lower than the skin covered with cotton.
J. Zhu's group, Nat. Nanotechnol., 2021[35]	Nanoprocessed silk with Al_2O_3 nanoparticles	Silk microfibers wrapped with Al_2O_3 nanoparticles with a size of 300 nm	202 µm	Under direct sunlight (peak solar irradiance > 900 W m^{-2}), the sample stage is covered with the fabric.	~3.5 °C below ambient
J. Zhu's group, Nat. Nanotechnol., 2021[36]	Electrospun polyethylene oxide film	Nanofibers with selective emissivity in TASW	500 µm	Under a peak solar intensity of near 900 W m^{-2}, the sample stage is covered with the film.	~5 °C below ambient
H. Zhu's group, ACS Appl. Mater. Interfaces, 2021[37]	Electrospun PVDF–Al_2O_3 film	Composite fibers (diameter 0.5–2.5 µm) consisting of PVDF matrix and Al_2O_3 particles (260–470 nm)	500 µm	Under an average solar intensity of 850 W m^{-2}, the sample stage is covered with the film.	~4 °C below ambient
G. Tao's group, Science, 2021[38]	TiO_2–polylactic acid (PLA)/ polytetrafluoroethylene (PTFE) textile	Multilayer TiO_2–PLA composite textile laminated with a thin PTFE layer	550 µm	Under a solar intensity of ~630 W m^{-2}, the sample stage is covered with the textile.	~2 °C below ambient
T. Fan's group, Adv. Funct. Mater., 2020[39]	PVDF–tetraethoxysilane (TEOS)/SiO_2 membrane	Nanoporous electrospun PVDF–TEOS fibers with SiO_2 microspheres distributed across their surfaces	300 µm	Under a solar intensity of ~1 kW m^{-2}, the sample stage is covered with the membrane.	Average 3.5 °C below ambient
A. Lenert's group, ACS Appl. Mater. Interfaces, 2020[40]	Electrospun polyacrylonitrile film	Bead-on-string fibers with ellipsoidal and cylindrical geometries	720 µm	Under a solar intensity of ~960 W m^{-2}, the sample stage is covered with the film.	3 °C below ambient
This work	3D EMPA-L made from PVDF	Electrospun micropyramid array	21.9 µm for the substrate, 24.75 µm for the average micropyramid height	Under a solar intensity of 1 kW m^{-2}, the sample stage is covered with the fabric.	3.8 °C below ambient
				The skin covered with different sample films is exposed to sunlight with an intensity of 1 kW m^{-2} for 8 min.	4.0 °C lower than the skin exposed to sunlight
					2.4 °C lower than the skin covered with a cotton-containing fabric.

The cooling temperature values marked in orange represent the data measured under intense solar radiation (solar irradiance > 600 W m^{-2}). The values marked in navy blue mean that the cooling temperatures are recorded without the influence of direct or intense sunlight.

Supplementary Table 5 Pressure sensing performance comparison among EMPA-based devices and other similar devices with different microstructures.

Ref.	Sample & structure	Feature size	Maximum sensitivity	Detection limit	Response time
T. Someya's group, Science, 2020[41]	Piezocapacitive sensor based on the flat electrospun parylene/polyurethane film	Nanofiber diameter 200–400 nm	0.141 kPa^{-1}	Not reported	190–220 ms
Z. Bao's group, Nat. Mater., 2010[42]	Piezocapacitive sensor based on the PDMS micropyramid array	Both size and pitch 6 μm	0.55 kPa^{-1}	3 Pa	Not reported
	Piezocapacitive sensor based the line-structured PDMS film	Both size and interval 6 μm	0.1 kPa^{-1}	Not reported	Not reported
S. Wang's group, Sci. Adv., 2021[43]	Piezocapacitive sensor based on the micropyramid-arrayed ionic elastomer.	Side length 500 μm, height 350 μm	4.5 kPa^{-1}	0.2 Pa	~50 ms
C. F. Guo's group, Nat. Commun., 2022[44]	Piezocapacitive sensor based on the PDMS-carbon nanotube microcone array	Bottom diameter 40 μm	0.15 kPa^{-1}	~0.35 Pa	6 ms
N. Lu's group, Adv. Mater., 2021[45]	Piezocapacitive sensor based on the carbon nanotubes–Ecoflex porous structure	Pore size several hundred microns	3.13 kPa^{-1}	0.07 Pa	94 ms
Z. Bao's group, Adv. Funct. Mater., 2020[24]	Piezocapacitive sensor based on the PDMS micropyramid array	Both size and interval 50 μm	0.022 kPa^{-1}	Not reported	Not reported
A. Khademhosseini's group, Adv. Funct. Mater., 2020[46]	Piezocapacitive sensor based on the gelatin methacryloyl micropyramid array	Side length 600 μm, height 420 μm, interval 1 mm	0.19 kPa^{-1}	0.1 Pa	161 ms
J. Y. Park's group, Adv. Funct. Mater., 2020[47]	Piezocapacitive sensor based on the $CaCu_3Ti_4O_{12}$–polyurethane microporous structure	Pore size ~0.2mm	0.73 kPa^{-1}	Not reported	84 ms
W. H. Lee's group, ACS Appl. Mater. Interfaces, 2020[48]	Piezocapacitive sensor based on the PDMS dome array	Both radius and height 5 μm	0.245 kPa^{-1}	1.44 Pa	300 ms
	Piezocapacitive sensor based on the PDMS square array	Both side length and height 5 μm	0.00144 kPa^{-1}	198 Pa	410 ms
C. F. Guo's group, Adv. Electron. Mater.,2018[49]	Piezocapacitive sensor based on the microtower array	Diameter 6.5 μm, height 14 μm	1.194 kPa^{-1}	< 0.8 Pa	36 ms
R. Sun, Chem. Eur.J., 2018[50]	Piezocapacitive sensor based on the line-structured polyurethane-AgNW composite film	Width of line structures ~1 μm	1.9 kPa^{-1}	10 Pa	100 ms
S. Park's group, ACS Appl. Mater. Interfaces, 2019[51]	Piezocapacitive sensor based on the highly ordered micropores	Pore size 500 μm	~0.8 kPa^{-1}	10 Pa	100 ms
T. Lee's group, Adv. Electron. Mater, 2016[52]	Piezocapacitive sensor based on the hierarchical porous structure	Pore size 6 μm	0.63 kPa^{-1}	2.42 Pa	~40 ms
Z. L. Wang's group, Sci. Adv., 2020[53]	Triboelectric sensor based on the textile consisting of nylon yarns, terylene–stainless steel conductive yarns, and clothing	Diameters of conductive yarn and nylon yarn 200 and 150 μm, respectively	7.84 V kPa^{-1}	Not reported	20 ms
Z. Du's group, Nano Energy, 2021[54]	Triboelectric sensor based on the multi-leveled structures as well as the wrinkled structures	Surface roughness 32.913 μm	0.293 V kPa^{-1}	0.23 Pa	26 ms
Z. Du's group, Nano Energy, 2018[55]	Piezocapacitive and triboelectric sensor based on the core-shell PDMS ion gel–PVDF-HFP nanofibers	Diameters of total nanofiber and core nanofiber 235 and 35 μm, respectively	0.43 kPa^{-1}	10 Pa	Not reported

X.-S. Zhang's group, Adv. Fiber Mater., 2022[56]	Piezocapacitive sensor based on the flat electrospun silk film	Nanofiber diameter 200–400 nm	0.075 kPa^{-1}	~26 Pa	Not reported
This work	EMPA piezocapacitive-triboelectric hybrid sensor	Average micropyramid height 24.75 and 18.23 μm	19 kPa^{-1}	0.05 Pa	0.8 ms

Supplementary Table 6 Energy harvesting performance comparison among the EMPA-based nanogenerators and other all-fiber and nanofiber-based nanogenerators.

Ref.	Device type	Main material	Working area [mm^2]	Force [N]	Maximum current density [$mA\,m^{-2}$]	Maximum voltage [V]	Transfer charge density [$\mu C\,m^{-2}$]
L. Wei's group, Nat. Commun., 2021[57]	TENG	Al and styrene-ethylene-butylene-styrene/GaIn alloy fibers	6 × 100 × 0.8	Not reported	0.014	1.25	Not reported
Z. L. Wang's group, Sci. Adv., 2021[58]	TENG	Polylactic-co-glycolic acid/Ag NW/PVA fabric and PTFE film	20 × 20	Not reported	~3.75	95	75
F. Sorin's group, Nat. Commun., 2020[59]	TENG	Geniomer/liquid metal fabric	60 × 60	~25.5	Not reported	~490	48.6
Z. L. Wang's group, Sci. Adv., 2016[60]	TENG	Cu-based and PDMS-based fabrics	100 × 100	Not reported	0.091	Not reported	2.08
B. Ding's group, Adv. Funct. Mater., 2020[8]	TENG	Electrospun PVDF/carbon/PU fabric	40 × 40	Not reported	Not reported	220	Not reported
P. S. Lee's group, Nano Energy, 2020[61]	TENG	Al and electrospun PVDF-HFP and styrene-ethylene-butylene-styrene fabrics	20 × 20	30	10	85	37.5
B. Ding's group, Nano Energy, 2017[62]	TENG	Electrospun PVDF/PDMS, PAN/polyamide-6 (PA6) fabrics	1600	100	68.8	250	~120
H. Fan's group, Nano Energy, 2017[63]	TENG	Al and electrospun PVDF/Fe_3O_4 fabric	55×30	15.17	4.24	98	Not reported
J. H. Oh's group, Appl. Phys. Lett., 2016[7]	TENG	Al and electrospun honeycomb-like P(VDF-TrFE) fabric	40 × 40	30	10.625	160	Not reported
H. Yu's group, Sci. Rep., 2015[64]	TENG	Electrospun poly(3-hydroxybutyrate-co-3-hydroxyvalerate) and PVDF–graphene oxide fabric	50 × 40	Not reported	39	340	Not reported
G.-G. Wang's group, Nano Energy, 2021[65]	TPNG	Electrospun PA66/MWCNT and PVDF fabrics	20 × 20	15	38.75	142	Not reported
Q. Zhang's group, Nano Energy, 2018[66]	TPNG	Electrospun silk and PVDF fabrics	40 × 20	25.7	15	500	Not reported
Q. Zhang's group, Nano Energy, 2015[67]	TPNG	Electrospun PVDF and conducting fabrics	60 × 50	Not reported	15	210	Not reported
Z.-Q. Feng's group, Adv. Mater., 2021[68]	PENG	Electrospun P(VDF-TrFE) fibers	20 × 10	~0.2	Not reported	2	Not reported
H. A. Sodano's group, Energy Environ. Sci., 2014[69]	PENG	Vertically aligned $BaTiO_3$ nanowire arrays	10 × 10	Not reported	0.009	0.312	Not reported
S.-W. Kuo's group, J. Mater. Chem. A, 2015[70]	PENG	Electrospun PVDF fibers	5 × 5	Not reported	Not reported	0.12	Not reported
Y. Ding's group, J. Mater. Chem. C, 2015[71]	PENG	Vertically aligned P(VDF-TrFE) nanowire arrays	10 × 10	4	Not reported	~1.8	Not reported

Y. Li's group, Sci. Rep., 2013[72]		Electrospun PVDF fibers	1962.5	39.25	Not reported	0.42	Not reported
H. Wang's group, Nanotechnology, 2013[73]		Electrospun PVDF fibers	80 × 80	Not reported	Not reported	2	Not reported
N. K. Lee's group, ACS Appl. Mater. Interfaces, 2016[74]		Electrospun P(VDF-TrFE) fibers	15 × 15	1.485	Not reported	1.96	Not reported
Y. Shi's group, Nano Lett., 2010[75]		Electrospun lead zirconate titanate fibers	Not reported	Not reported	Not reported	1.6	Not reported
J. A. Rogers's group, Nat. Commun., 2013[76]		Electrospun P(VDF-TrFE) fibers	25 × 8	Not reported	0.2	1.5	Not reported
Y. Jiang's group, ACS Appl. Mater. Interfaces, 2019[77]		Electrospun P(VDF-TrFE)/$BaTiO_3$ fibers	64	~11	0.195	Not reported	0.544
This work	PENG	3D EMPA-L	20 × 20	5	0.485	6.8	1.719
	TENG	Electrospun PA66 fabric and depolarized 3D EMPA-M	45 × 45	5	27.51	784.7	51.48
				15	44.95	1160	86.99
				30	51.74	1300	105.1
		Au-coated 2D EP and depolarized 3D EMPA-M	20 × 20	5	6.469	32.06	15.70

Supplementary References

1. Cheng, W., Wang J., Ma Z., Yan K., Wang Y., Wang H., Li S., Li Y., Pan L. & Shi Y. Flexible pressure sensor with high sensitivity and low hysteresis based on a hierarchically microstructured electrode. *IEEE Electron Device Lett.* **39**, 288–291 (2018).
2. Yang, W., Gong W., Hou C., Su Y., Guo Y., Zhang W., Li Y., Zhang Q. & Wang H. All-fiber tribo-ferroelectric synergistic electronics with high thermal-moisture stability and comfortability. *Nat. Commun.* **10**, 5541 (2019).
3. Song, Y., Shi Z., Hu G.-H., Xiong C., Isogai A. & Yang Q. Recent advances in cellulose-based piezoelectric and triboelectric nanogenerators for energy harvesting: A review. *J. Mater. Chem. A* **9**, 1910–1937 (2021).
4. Zhang, J.-H., Li Y., Du J., Hao X. & Wang Q. Bio-inspired hydrophobic/cancellous/hydrophilic trimurti pvdf mat-based wearable triboelectric nanogenerator designed by self-assembly of electro-pore-creating. *Nano Energy* **61**, 486–495 (2019).
5. Zhang, J.-H., Li Y., Du J., Hao X. & Huang H. A high-power wearable triboelectric nanogenerator prepared from self-assembled electrospun poly(vinylidene fluoride) fibers with a heart-like structure. *J. Mater. Chem. A* **7**, 11724–11733 (2019).
6. Zhang, J.-H., Li Y. & Hao X. A high-performance triboelectric nanogenerator with improved output stability by construction of biomimetic superhydrophobic nanoporous fibers. *Nanotechnology* **31**, 215401 (2020).
7. Jang, S., Kim H., Kim Y., Kang B. J. & Oh J. H. Honeycomb-like nanofiber based triboelectric nanogenerator using self-assembled electrospun poly(vinylidene fluoride-co-trifluoroethylene) nanofibers. *Appl. Phys. Lett.* **108**, 143901 (2016).

8. Li, Z., Zhu M., Shen J., Qiu Q., Yu J. & Ding B. All-fiber structured electronic skin with high elasticity and breathability. *Adv. Funct. Mater.* **30**, 1908411 (2020).

9. Meng, B., Tang W., Too Z.-h., Zhang X., Han M., Liu W. & Zhang H. A transparent single-friction-surface triboelectric generator and self-powered touch sensor. *Energy Environ. Sci.* **6**, 3235–3240 (2013).

10. Zhang, X.-S., Han M.-D., Wang R.-X., Meng B., Zhu F.-Y., Sun X.-M., Hu W., Wang W., Li Z.-H. & Zhang H.-X. High-performance triboelectric nanogenerator with enhanced energy density based on single-step fluorocarbon plasma treatment. *Nano Energy* **4**, 123–131 (2014).

11. Sajkiewicz, P. Crystallization behaviour of poly(vinylidene fluoride). *Eur. Polym. J.* **35**, 1581–1590 (1999).

12. Mohammadi, B., Yousefi A. A. & Bellah S. M. Effect of tensile strain rate and elongation on crystalline structure and piezoelectric properties of pvdf thin films. *Polym. Test.* **26**, 42–50 (2007).

13. Taylor, G. Disintegration of water drops in an electric field. *Proc. R. Soc. Lond. A* **280**, 383–397 (1964).

14. Ahirwal, D., Hébraud A., Kádár R., Wilhelm M. & Schlatter G. From self-assembly of electrospun nanofibers to 3d cm thick hierarchical foams. *Soft Matter* **9**, 3164–3172 (2013).

15. Hohman, M. M., Shin M., Rutledge G. & Brenner M. P. Electrospinning and electrically forced jets. I. Stability theory. *Phys. Fluids* **13**, 2201–2220 (2001).

16. Li, D., Wang Y. & Xia Y. Electrospinning of polymeric and ceramic nanofibers as uniaxially aligned arrays. *Nano Lett.* **3**, 1167–1171 (2003).

17. Yan, G., Yu J., Qiu Y., Yi X., Lu J., Zhou X. & Bai X. Self-assembly of electrospun polymer nanofibers: A general phenomenon generating honeycomb-patterned nanofibrous structures. *Langmuir* **27**, 4285–4289 (2011).

18. Reis, T. C., Correia I. J. & Aguiar-Ricardo A. Electrodynamic tailoring of self-assembled three-dimensional electrospun constructs. *Nanoscale* **5**, 7528–7536 (2013).

19. Sun, B., Long Y. Z., Yu F., Li M. M., Zhang H. D., Li W. J. & Xu T. X. Self-assembly of a three-dimensional fibrous polymer sponge by electrospinning. *Nanoscale* **4**, 2134–2137 (2012).

20. Deitzel, J. M., Kleinmeyer J., Harris D. & Tan N. C. B. The effect of processing variables on the morphology of electrospun nanofibers and textiles. *Polymer* **42**, 261–272 (2001).

21. Experimental characterization of electrospinning: The electrically forced jet and instabilities. *Polymer* **42**, 9955−9967 (2001).

22. Roy, K.-M. Sulfones and sulfoxides. *Ullmann's Encyclopedia of Industrial Chemistry* **34**, 705–720 (2000).

23. Zhang, H., Ly K. C. S., Liu X., Chen Z., Yan M., Wu Z., Wang X., Zheng Y., Zhou H. & Fan T. Biologically inspired flexible photonic films for efficient passive radiative cooling. *Proc. Natl. Acad. Sci. U.S.A.* **117**, 14657–14666 (2020).

24. Ruth, S. R. A., Beker L., Tran H., Feig V. R., Matsuhisa N. & Bao Z. Rational design of capacitive pressure sensors based on pyramidal microstructures for specialized monitoring of biosignals. *Adv. Funct. Mater.* **30**, 1903100 (2019).

25. Tee, B. C. K., Chortos A., Dunn R. R., Schwartz G., Eason E. & Bao Z. Tunable flexible pressure sensors using microstructured elastomer geometries for intuitive electronics. *Adv. Funct. Mater.* **24**, 5427–5434 (2014).

26. Ruth, S. R. A. & Bao Z. Designing tunable capacitive pressure sensors based on material properties and microstructure geometry. *ACS Appl. Mater. Interfaces* **12**, 58301–58316 (2020).

27. Fan, F. R., Lin L., Zhu G., Wu W., Zhang R. & Wang Z. L. Transparent triboelectric nanogenerators and self-powered pressure sensors based on micropatterned plastic films. *Nano Lett.* **12**, 3109–3114 (2012).

28. Ahmed, A., Hassan I. & Zu J. Design guidelines of stretchable pressure sensors-based triboelectrification. *Adv. Eng. Mater.* **20**, (2018).

29. Zhou, Q., Park J. G., Kim K. N., Thokchom A. K., Bae J., Baik J. M. & Kim T. Transparent-flexible-multimodal triboelectric nanogenerators for mechanical energy harvesting and self-powered sensor applications. *Nano Energy* **48**, 471–480 (2018).

30. Lee, J.-H., Yoon H.-J., Kim T. Y., Gupta M. K., Lee J. H., Seung W., Ryu H. & Kim S.-W. Micropatterned p(vdf-trfe) film-based piezoelectric nanogenerators for highly sensitive self-powered pressure sensors. *Adv. Funct. Mater.* **25**, 3203–3209 (2015).

31. Zhu, G., Zhou Y. S., Bai P., Meng X. S., Jing Q., Chen J. & Wang Z. L. A shape-adaptive thin-film-based approach for 50% high-efficiency energy generation through micro-grating sliding electrification. *Adv. Mater.* **26**, 3788–3796 (2014).

32. Ortega, R., Hansen C. J., Elterman K. & Woo A. Pulse oximetry. *N. Engl. J. Med.* **364**, e33 (2011).

33. Hsu, P.-C., Song A. Y., Catrysse P. B., Liu C., Peng Y., Xie J., Fan S. & Cui Y. Radiative human body cooling by nanoporous polyethylene textile. *Science* **353**, 1019–1023 (2016).

34. Peng, Y., Chen J., Song A. Y., Catrysse P. B., Hsu P.-C., Cai L., Liu B., Zhu Y., Zhou G., Wu D. S., Lee H. R., Fan S. & Cui Y. Nanoporous polyethylene microfibres for large-scale radiative cooling fabric. *Nat. Sustain.* **1**, 105–112 (2018).

35. Zhu, B., Li W., Zhang Q., Li D., Liu X., Wang Y., Xu N., Wu Z., Li J., Li X., Catrysse P. B., Xu W., Fan S. & Zhu J. Subambient daytime radiative cooling textile based on nanoprocessed silk. *Nat. Nanotechnol.* **16**, 1342–1348 (2021).

36. Li, D., Liu X., Li W., Lin Z., Zhu B., Li Z., Li J., Li B., Fan S., Xie J. & Zhu J. Scalable and hierarchically designed polymer film as a selective thermal emitter for high-performance all-day radiative cooling. *Nat. Nanotechnol.* **16**, 153–158 (2021).

37. Jing, W., Zhang S., Zhang W., Chen Z., Zhang C., Wu D., Gao Y. & Zhu H. Scalable and flexible electrospun film for daytime subambient radiative cooling. *ACS Appl. Mater. Interfaces*, 29558–29566 (2021).

38. Zeng, S., Pian S., Su M., Wang Z., Wu M., Liu X., Chen M., Xiang Y., Wu J., Zhang M., Cen Q., Tang Y., Zhou X., Huang Z., Wang R., Tunuhe A., Sun X., Xia Z., Tian M., Chen M., Ma X., Yang L., Zhou J., Zhou H., Yang Q., Li X., Ma Y. & Tao G. Hierarchical-morphology metafabric for scalable passive daytime radiative cooling. *Science* **373**, 692–696 (2021).

39. Wang, X., Liu X., Li Z., Zhang H., Yang Z., Zhou H. & Fan T. Scalable flexible hybrid membranes with photonic structures for daytime radiative cooling. *Adv. Funct. Mater.* **30**, 1907562 (2019).

40. Kim, H., McSherry S., Brown B. & Lenert A. Selectively enhancing solar scattering for direct radiative cooling through control of polymer nanofiber morphology. *ACS Appl. Mater. Interfaces* **12**, 43553–43559 (2020).

41. Lee, S., Franklin S., Hassani F. A., Yokota T., Nayeem M. O. G., Wang Y., Leib R., Cheng G., Franklin D. W. & Someya T. Nanomesh pressure sensor for monitoring finger manipulation without sensory interference. *Science* **370**, 966–970 (2020).

42. Mannsfeld, S. C., Tee B. C., Stoltenberg R. M., Chen C. V., Barman S., Muir B. V., Sokolov A. N., Reese C. & Bao Z. Highly sensitive flexible pressure sensors with microstructured rubber dielectric layers. *Nat. Mater.* **9**, 859–864 (2010).

43. Su, Q., Zou Q., Li Y., Chen Y., Teng S.-Y., Kelleher J. T., Nith R., Cheng P., Li N., Liu W., Dai S., Liu Y., Mazursky A., Xu J., Jin L., Lopes P. & Wang S. A stretchable

and strain-unperturbed pressure sensor for motion interference–free tactile monitoring on skins. *Sci. Adv.* **7**, eabi4563 (2021).

44. Zhang, Y., Yang J., Hou X., Li G., Wang L., Bai N., Cai M., Zhao L., Wang Y., Zhang J., Chen K., Wu X., Yang C., Dai Y., Zhang Z. & Guo C. F. Highly stable flexible pressure sensors with a quasi-homogeneous composition and interlinked interfaces. *Nat. Commun.* **13**, 1317 (2022).

45. Ha, K. H., Zhang W., Jang H., Kang S., Wang L., Tan P., Hwang H. & Lu N. Highly sensitive capacitive pressure sensors over a wide pressure range enabled by the hybrid responses of a highly porous nanocomposite. *Adv. Mater.* **33**, e2103320 (2021).

46. Li, Z., Zhang S., Chen Y., Ling H., Zhao L., Luo G., Wang X., Hartel M. C., Liu H., Xue Y., Haghniaz R., Lee K., Sun W., Kim H., Lee J., Zhao Y., Zhao Y., Emaminejad S., Ahadian S., Ashammakhi N., Dokmeci M. R., Jiang Z. & Khademhosseini A. Gelatin methacryloyl-based tactile sensors for medical wearables. *Adv. Funct. Mater.* **30**, (2020).

47. Chhetry, A., Sharma S., Yoon H., Ko S. & Park J. Y. Enhanced sensitivity of capacitive pressure and strain sensor based on cacu3ti4o12 wrapped hybrid sponge for wearable applications. *Adv. Funct. Mater.* **30**, 1910020 (2020).

48. Hwang, J., Lee S. G., Kim S., Kim J. S., Kim D. H. & Lee W. H. Unveiling viscoelastic response of capacitive-type pressure sensor by controlling cross-linking density and surface structure of elastomer. *ACS Appl. Polym. Mater.* **2**, 2190–2198 (2020).

49. Wan, Y., Qiu Z., Hong Y., Wang Y., Zhang J., Liu Q., Wu Z. & Guo C. F. A highly sensitive flexible capacitive tactile sensor with sparse and high-aspect-ratio microstructures. *Adv. Electron. Mater.* **4**, 1700586 (2018).

50. Liu, F., Han F., Ling L., Li J., Zhao S., Zhao T., Liang X., Zhu D., Zhang G., Sun R., Ho D. & Wong C. P. An omni-healable and highly sensitive capacitive pressure sensor with microarray structure. *Chemistry* **24**, 16823-16832 (2018).

51. Kim, J. O., Kwon S. Y., Kim Y., Choi H. B., Yang J. C., Oh J., Lee H. S., Sim J. Y., Ryu S. & Park S. Highly ordered 3d microstructure-based electronic skin capable of differentiating pressure, temperature, and proximity. *ACS Appl. Mater. Interfaces* **11**, 1503–1511 (2019).

52. Kang, S., Lee J., Lee S., Kim S., Kim J.-K., Algadi H., Al-Sayari S., Kim D.-E., Kim D. & Lee T. Highly sensitive pressure sensor based on bioinspired porous structure for real-time tactile sensing. *Adv. Electron. Mater.* **2**, 1600356 (2016).

53. Fan, W., He Q., Meng K., Tan X., Zhou Z., Zhang G., Yang J. & Wang Z. L. Machine-knitted washable sensor array textile for precise epidermal physiological signal monitoring. *Sci. Adv.* **6**, eaay2840 (2020).

54. Wang, J., Cui P., Zhang J., Ge Y., Liu X., Xuan N., Gu G., Cheng G. & Du Z. A stretchable self-powered triboelectric tactile sensor with egain alloy electrode for ultra-low-pressure detection. *Nano Energy* **89**, 106320 (2021).

55. Lin, M.-F., Xiong J., Wang J., Parida K. & Lee P. S. Core-shell nanofiber mats for tactile pressure sensor and nanogenerator applications. *Nano Energy* **44**, 248–255 (2018).

56. Wen, D.-L., Pang Y.-X., Huang P., Wang Y.-L., Zhang X.-R., Deng H.-T. & Zhang X.-S. Silk fibroin-based wearable all-fiber multifunctional sensor for smart clothing. *Adv. Fiber Mater.* **4**, 873–884 (2022).

57. Chen, M., Wang Z., Zhang Q., Wang Z., Liu W., Chen M. & Wei L. Self-powered multifunctional sensing based on super-elastic fibers by soluble-core thermal drawing. *Nat. Commun.* **12**, 1416 (2021).

58. Peng, X., Dong K., Ye C., Jiang Y., Zhai S., Cheng R., Liu D., Gao X., Wang J. & Wang Z. L. A breathable, biodegradable, antibacterial, and self-powered electronic skin based on all-nanofiber triboelectric nanogenerators. *Sci. Adv.* **6**, eaba9624 (2020).

59. Dong, C., Leber A., Das Gupta T., Chandran R., Volpi M., Qu Y., Nguyen-Dang T., Bartolomei N., Yan W. & Sorin F. High-efficiency super-elastic liquid metal based triboelectric fibers and textiles. *Nat. Commun.* **11**, 3537 (2020).

60. Zhen Wen, Min-Hsin Yeh, Hengyu Guo, Jie Wang, Yunlong Zi, Weidong Xu, Jianan Deng, Lei Zhu, Xin Wang, Chenguo Hu, Liping Zhu, Xuhui Sun & Wang Z. L. Self-powered textile for wearable electronics by hybridizing fiber-shaped nanogenerators, solar cells, and supercapacitors. *Sci. Adv.* **2**, e1600097 (2016).

61. Li, Y., Xiong J., Lv J., Chen J., Gao D., Zhang X. & Lee P. S. Mechanically interlocked stretchable nanofibers for multifunctional wearable triboelectric nanogenerator. *Nano Energy* **78**, 105358 (2020).

62. Li, Z., Shen J., Abdalla I., Yu J. & Ding B. Nanofibrous membrane constructed wearable triboelectric nanogenerator for high performance biomechanical energy harvesting. *Nano Energy* **36**, 341–348 (2017).

63. Ren, X., Fan H., Wang C., Ma J., Lei S., Zhao Y., Li H. & Zhao N. Magnetic force driven noncontact electromagnetic-triboelectric hybrid nanogenerator for scavenging biomechanical energy. *Nano Energy* **35**, 233–241 (2017).

64. Huang, T., Lu M., Yu H., Zhang Q., Wang H. & Zhu M. Enhanced power output of a triboelectric nanogenerator composed of electrospun nanofiber mats doped with graphene oxide. *Sci. Rep.* **5**, 13942 (2015).

65. Sun, N., Wang G.-G., Zhao H.-X., Cai Y.-W., Li J.-Z., Li G.-Z., Zhang X.-N., Wang B.-L., Han J.-C., Wang Y. & Yang Y. Waterproof, breathable and washable triboelectric nanogenerator based on electrospun nanofiber films for wearable electronics. *Nano Energy* **90**, 106639 (2021).

66. Guo, Y., Zhang X.-S., Wang Y., Gong W., Zhang Q., Wang H. & Brugger J. All-fiber hybrid piezoelectric-enhanced triboelectric nanogenerator for wearable gesture monitoring. *Nano Energy* **48**, 152–160 (2018).

67. Huang, T., Wang C., Yu H., Wang H., Zhang Q. & Zhu M. Human walking-driven wearable all-fiber triboelectric nanogenerator containing electrospun polyvinylidene fluoride piezoelectric nanofibers. *Nano Energy* **14**, 226–235 (2015).

68. Li, T., Qu M., Carlos C., Gu L., Jin F., Yuan T., Wu X., Xiao J., Wang T., Dong W., Wang X. & Feng Z. Q. High-performance poly(vinylidene difluoride)/dopamine core/shell piezoelectric nanofiber and its application for biomedical sensors. *Adv. Mater.* **33**, e2006093 (2021).

69. Koka, A., Zhou Z. & Sodano H. A. Vertically aligned batio3nanowire arrays for energy harvesting. *Energy Environ. Sci.* **7**, 288–296 (2014).

70. Pan, C.-T., Yen C.-K., Wu H.-C., Lin L., Lu Y.-S., Huang J. C.-C. & Kuo S.-W. Significant piezoelectric and energy harvesting enhancement of poly(vinylidene fluoride)/polypeptide fiber composites prepared through near-field electrospinning. *J. Mater. Chem. A* **3**, 6835–6843 (2015).

71. Chen, X., Shao J., An N., Li X., Tian H., Xu C. & Ding Y. Self-powered flexible pressure sensors with vertically well-aligned piezoelectric nanowire arrays for monitoring vital signs. *J. Mater. Chem. C* **3**, 11806–11814 (2015).

72. Hou, C., Huang T., Wang H., Yu H., Zhang Q. & Li Y. A strong and stretchable self-healing film with self-activated pressure sensitivity for potential artificial skin applications. *Sci. Rep.* **3**, 3138 (2013).

73. Yu, H., Huang T., Lu M., Mao M., Zhang Q. & Wang H. Enhanced power output of an electrospun pvdf/mwcnts-based nanogenerator by tuning its conductivity. *Nanotechnology* **24**, 405401 (2013).

74. Park, S. H., Lee H. B., Yeon S. M., Park J. & Lee N. K. Flexible and stretchable piezoelectric sensor with thickness-tunable configuration of electrospun nanofiber mat and elastomeric substrates. *ACS Appl. Mater. Interfaces* **8**, 24773–24781 (2016).
75. Chen, X., Xu S., Yao N. & Shi Y. 1.6 v nanogenerator for mechanical energy harvesting using pzt nanofibers. *Nano Lett.* **10**, 2133–2137 (2010).
76. Persano, L., Dagdeviren C., Su Y., Zhang Y., Girardo S., Pisignano D., Huang Y. & Rogers J. A. High performance piezoelectric devices based on aligned arrays of nanofibers of poly(vinylidenefluoride-co-trifluoroethylene). *Nat. Commun.* **4**, 1633 (2013).
77. Hu, X., Yan X., Gong L., Wang F., Xu Y., Feng L., Zhang D. & Jiang Y. Improved piezoelectric sensing performance of p(vdf-trfe) nanofibers by utilizing bto nanoparticles and penetrated electrodes. *ACS Appl. Mater. Interfaces* **11**, 7379-7386 (2019).
